# Identification of the first structurally validated covalent ligands of the small GTPase RAB27A†

**DOI:** 10.1039/d1md00225b

**Published:** 2021-12-16

**Authors:** Mostafa Jamshidiha, Thomas Lanyon-Hogg, Charlotte L. Sutherell, Gregory B. Craven, Montse Tersa, Elena De Vita, Delia Brustur, Inmaculada Pérez-Dorado, Sarah Hassan, Rita Petracca, Rhodri M. Morgan, Máximo Sanz-Hernández, Jim C. Norman, Alan Armstrong, David J. Mann, Ernesto Cota, Edward W. Tate

**Affiliations:** Department of Life Sciences, Imperial College London London SW7 2AZ UK e.cota@imperial.ac.uk; Department of Chemistry, Imperial College London London W12 0BZ UK e.tate@imperial.ac.uk; Beatson Institute for Cancer Research, Garscube Estate Glasgow G61 1BD UK

## Abstract

Rab27A is a small GTPase, which mediates transport and docking of secretory vesicles at the plasma membrane *via* protein–protein interactions (PPIs) with effector proteins. Rab27A promotes the growth and invasion of multiple cancer types such as breast, lung and pancreatic, by enhancing secretion of chemokines, metalloproteases and exosomes. The significant role of Rab27A in multiple cancer types and the minor role in adults suggest that Rab27A may be a suitable target to disrupt cancer metastasis. Similar to many GTPases, the flat topology of the Rab27A-effector PPI interface and the high affinity for GTP make it a challenging target for inhibition by small molecules. Reported co-crystal structures show that several effectors of Rab27A interact with the Rab27A SF4 pocket (‘WF-binding pocket’) *via* a conserved tryptophan–phenylalanine (WF) dipeptide motif. To obtain structural insight into the ligandability of this pocket, a novel construct was designed fusing Rab27A to part of an effector protein (fRab27A), allowing crystallisation of Rab27A in high throughput. The paradigm of KRas covalent inhibitor development highlights the challenge presented by GTPase proteins as targets. However, taking advantage of two cysteine residues, C123 and C188, that flank the WF pocket and are unique to Rab27A and Rab27B among the >60 Rab family proteins, we used the quantitative Irreversible Tethering (qIT) assay to identify the first covalent ligands for native Rab27A. The binding modes of two hits were elucidated by co-crystallisation with fRab27A, exemplifying a platform for identifying suitable lead fragments for future development of competitive inhibitors of the Rab27A-effector interaction interface, corroborating the use of covalent libraries to tackle challenging targets.

## Abstract 2

A novel Rab27A construct enables elucidation of covalent ligand binding, paving the way for structure-guided approaches against this challenging target.

## Graphical abstract

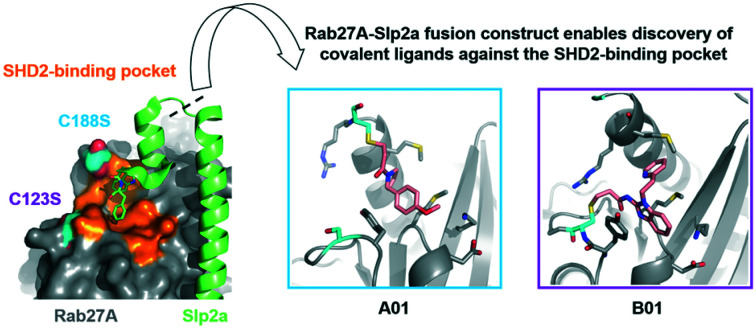

## Introduction

Rab proteins are a family of over 60 small guanosine triphosphate hydrolases (GTPases) involved in diverse intracellular membrane trafficking and vesicle transport processes, and are implicated in several disease states.^1^ As GTPases, Rab proteins cycle between an inactive, GDP-bound conformation and an active, GTP-bound conformation through the action of GTPase activating proteins (GAPs) and guanine-nucleotide exchange factors (GEFs). GTP-Bound Rab recruits effector proteins through protein–protein interactions (PPIs) to mediate trafficking.^2^ Despite their high interest as drug targets, the flat topology of GTPase PPI interfaces and high affinity for nucleotide binding make them challenging targets for small molecule inhibition, with stapled peptides being the only validated Rab ligands to-date, for Rab8A and Rab25.^3,4^

Rab27A and Rab27B are secretory Rabs with well-characterised roles in transport and docking of secretory vesicles (exosomes) at the plasma membrane.^5^ Aberrant activity of the export machinery in cancer cells results in release of specific regulatory proteins and microRNAs through exosome secretion.^6^ Very recently Rab27A was shown to recruit both SPIRE-type actin filament nucleators and melanophilin/myosin Va to melanosome organelles in melanocytes, thus converging both actin track assembly and motor activity at vesicle membranes.^7^ Rab27A promotes formation of pre-metastatic niches *via* exosome release and recycling of transmembrane proteins, including matrix metalloprotease MT1-MMP.^8^ Upregulation of Rab27A, for example in response to extracellular glutamate, drives invasive behaviour in aggressive cancers.^8^ Indeed, Rab27A is overexpressed in cancers of the breast, lung, and pancreas,^9–11^ and is associated with poor prognosis and metastasis.^12^ Rab27A knockout mice (*Ashen*) and Rab27A/Rab27B double knockout mice are viable and have a moderate phenotype,^13^ whilst in humans loss-of-function mutations in Rab27A cause Griscelli syndrome type II, which is characterised by hypopigmentation and compromised immune development.^14^ Rab27A is therefore an interesting and novel target to disrupt cancer metastasis. Interest in therapeutic targeting of Rab27A has driven efforts to identify small molecule inhibitors;^15–18^ however, to-date putative ligands have not been validated by direct binding assays or structural studies.

In order to address the lack of well-validated chemical tools for Rab27A, we report here the first platform allowing structural studies of Rab27A ligands at atomic resolution, enabling fragment-based screening and discovery of the first structurally validated small molecule ligands of Rab27A.

## Results and discussion

**Fig. 1:**
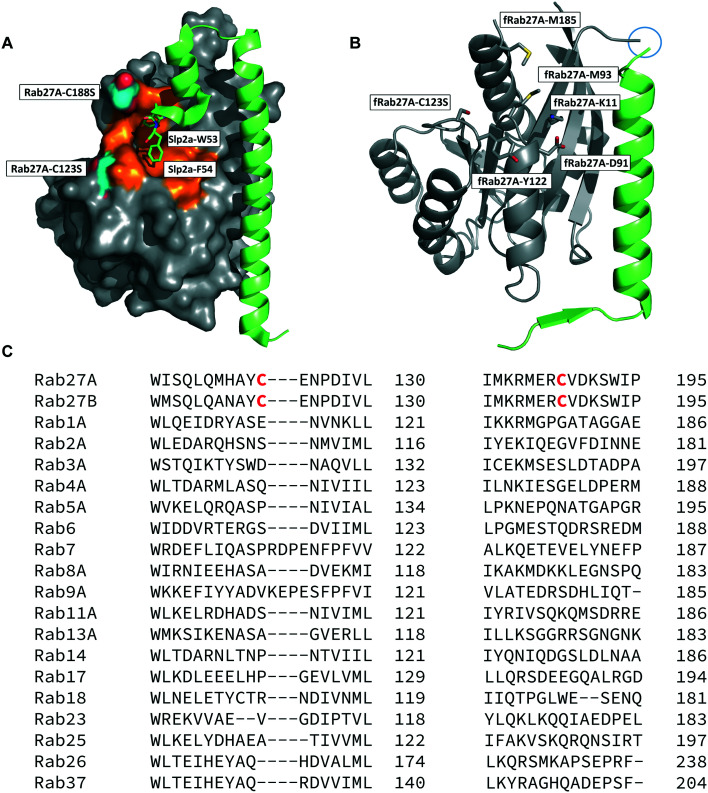
Rab27A-Slp2a and fRab27A structures for ligand discovery. A) Crystal structure (PDB: 3BC1) of the complex formed by Rab27A (grey) and the SHD1 and SHD2 domains of effector protein Exophilin4/Slp2a (green). The proposed ligandable Rab27A SF4 pocket (orange) is flanked by native cysteine 188 and cysteine 123, which were both mutated to serine for crystallographic purposes (cyan). B) Rab27A-Spl2-SHD1 fusion construct (fRab27A, PDB: 7OPP, Rab27A in grey and Spl2-SHD1 in green). C) Sequences of Rab27A and Rab27B aligned against a selection of closest homologues from the Rab family of proteins; C123 and C188 residues are unique to the Rab27 isoforms (highlighted in red). Full sequence alignment for the whole Rab family is shown in Fig. S4.†

The crystal structure of human Rab27A has been solved in complex with the Rab27-binding domain of the effector protein Exophilin4/Slp2a,^19^ showing a large interaction surface with the long α-helix of the effector synaptotagmin homology domain 1 (SHD1) packing against the hydrophobic surface of Rab27A. Rab proteins can be differentiated by four regions (SF1–SF4).^20^ The effector SHD2 domain interacts *via* a conserved tryptophan–phenylalanine (WF) dipeptide motif (residues 53–54) with the Rab27A SF4 region *via* π-stacking with tyrosine 122 (Y122) (Fig. 1A).^19^ This interaction is not conserved in GTPases other than Rab27A and Rab27B, suggesting this site may allow selective targeting. *In silico* analysis of the Rab27A surface for ligandable sites highlighted two major regions, the nucleotide pocket, and the WF dipeptide binding pocket at the effector binding interface (Fig. S1†). The GTPase nucleotide pocket has high affinity for GTP/GDP and conservation across the family, making it a very challenging site for selective small molecule inhibition. However, effector binding to Rab27A is strongly inhibited upon mutation of Y122 in the SF4 pocket,^19^ supporting the Rab27A WF-binding pocket as a potentially ligandable site suited to targeting Rab27-effector PPIs.

Structure-guided inhibitor development can be a powerful tool in targeting GTPases;^21^ however, in line with previous reports^19^ we were unable to crystallise monomeric Rab27A without substantial mutation to the PPI interface.^22^ We therefore designed a novel Rab27A construct to allow crystallisation whilst leaving the WF-binding pocket free for ligand binding. The C-terminus of Slp2a SHD1 (SFLTEEEQEAIMKVLQRDAALKRAEEER (residues 5–32)) was linked to the N-terminus of Rab27A *via* a flexible poly glycine-serine linker. The unstructured C-terminal hypervariable region of Rab27A was truncated and a Q78L mutation was introduced in the nucleotide binding pocket to promote adoption of the active GTP-bound conformation. We termed this class of constructs fusion-Rab27A (fRab27A); variants bearing C123S and C188S mutations (fRab27A), as well as single mutants specified by the presence of the wild-type cysteine at position 123 (fRab27A-C123) or 188 (fRab27A-C188), could be purified to homogeneity (Fig. S2†) and form protein crystals which diffract to high resolution (C123S, C188S, 2.32 Å, Table S1†). fRab27A adopted a closely similar conformation to the Rab27A-Slp2a co-crystal structure (Fig. 1B), with average root-mean-square deviation (RMSD) of 0.554 Å. Importantly, the structure leaves the WF-binding pocket unoccupied, as designed.

We used variants of this construct to undertake several ligand discovery campaigns, including fragment screens by differential scanning fluorimetry (DSF), nuclear magnetic resonance (NMR) and surface plasmon resonance (SPR), designed peptide fragments or peptidomimetics, and *in silico* modelling to design libraries complementary to the SF4 pocket. However, it proved extremely difficult to identify hits which could be co-crystallised in the WF-binding pocket of fRab27A, suggesting that this is a challenging pocket for small molecule ligands; a full account detailing these efforts will be reported in due course. Two compounds previously claimed as Rab27A ligands were also investigated, including BMD-11 (ref. 16) and Nexinhib-20 (ref. 15) (Fig. S3†). Both of these ligands were proposed to bind to the Rab27A WF-binding pocket from *in silico* studies; however, in these prior reports neither ligand was shown to bind Rab27A by any biochemical, biophysical or structural assay. Although the SF4 pocket had been speculatively proposed as the site of binding, neither of these ligands could be crystallised in the WF-binding pocket of fRab27A, nor could Rab27A binding be validated in biophysical assays (*e.g.* SPR). Collectively these studies demonstrated that, in-line with other GTPases, liganding of Rab27A remains challenging with small molecules.

**Fig. 2:**
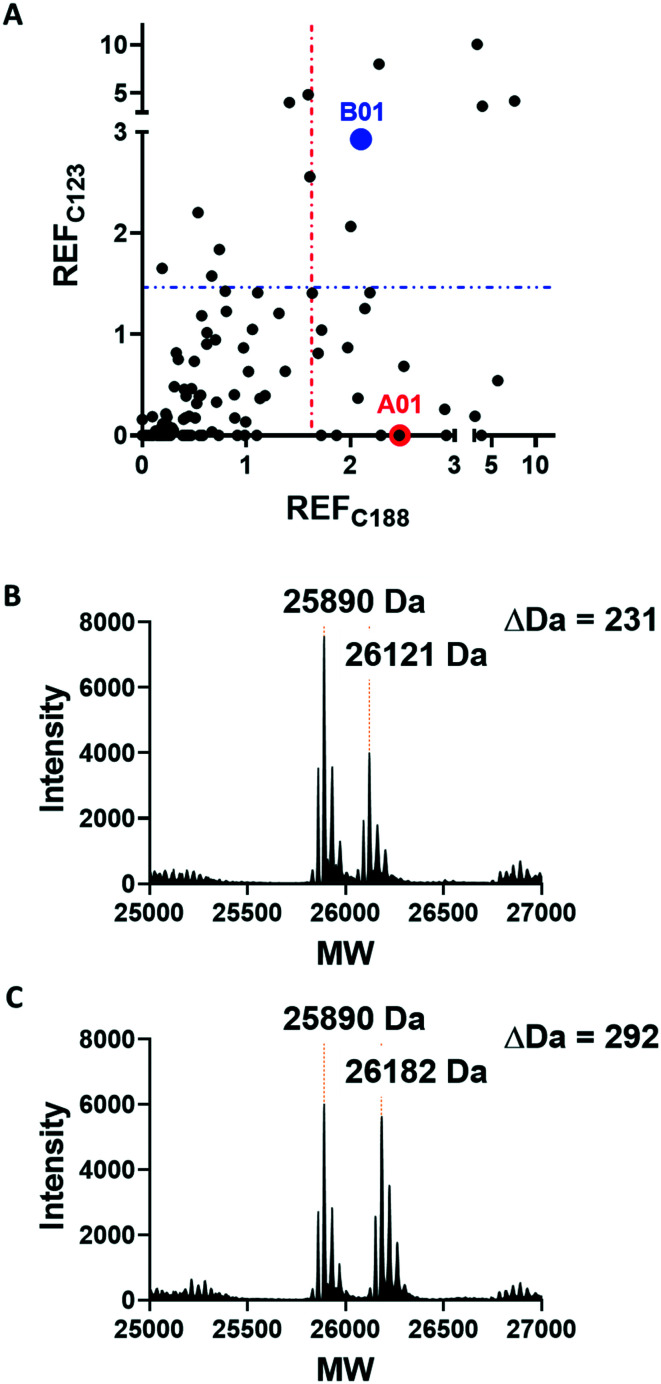
Acrylamide fragment screening against fRab27A-C123 and fRab27A-C188. A) 2D scatter plot of REF values against fRab27A-C123 (*Y*-axis) and fRab27A-C188 (*X*-axis) for 126-fragment library screened in the qIT assay. Hits were defined as REF values over one standard deviation from the median value, shown by a dotted line (in blue for fRab27A-C123, in red for fRab27A-C188). Selected hits A01 for fRab27A-C188 (red) and B01 for fRab27A-C123 (blue), are highlighted. B) Intact protein mass spectrometry for the fRab27A-C188-A01 adduct shows the expected mass shift (24 h incubation at 4 °C). C) Intact protein mass spectrometry for fRab27A-C123-B01 shows the expected mass shift (8 h incubation at 4 °C).

Covalent ligands are increasingly used to target challenging proteins.^23–26^ fRab27A contains two cysteine residues (C123 and C188) in close proximity to the WF-binding pocket, which are mutated to serine in crystal structures (Fig. 1A and B). Sequence alignment of Rab27A and Rab27B against the Rab family demonstrated that these two cysteine residues are unique to Rab27 among other family members (Fig. 1C and S4†), suggesting that cysteine-reactive covalent fragments targeting the WF binding site may offer a means to ligand Rab27 selectively over other Rabs. We expressed and purified mono-cysteine fRab27A constructs (Fig. S2†), fRab27A-C123 and fRab27A-C188 (where the residue number denotes the wild type cysteine not mutated in the construct) for fragment screening by our previously reported “quantitative Irreversible Tethering” (qIT) assay.^27^ The qIT assay measures the rate of modification of a thiol group in a protein or small molecule through quenching reaction aliquots with the fluorogenic dye 7-diethylamino-3-(4-maleimido-phenyl)-4-methylcoumarin (CPM) at a series of time points following addition of a putative electrophilic ligand.^27^ The decrease in fluorescence signal in sequential sample analysis over time reflects progressive covalent modification of the free Cys residue, and allows the rate of modification by an electrophilic fragment to be calculated. In a proof-of-concept pilot scale screen, a library of 126 fragments,^27^ each bearing an electrophilic acrylamide warhead, was screened against fRab27A-C123 and fRab27A-C188. To account for differences in acrylamide electrophilicity, rate constants were normalised to the rate constant for reaction with glutathione (GSH), generating a rate-enhancement factor (REF) over GSH for each fragment against each construct. Hits were defined as fragments with REF values over one standard deviation from the median value (Fig. 2A). Hit validation was performed by measurement of modification by intact-protein mass spectrometry at the timeframe required for 50% labelling from the qIT screen (Fig. 2B and C). At this selection stage, both the shift in the protein mass and the expected half-life of adduct formation were evaluated for consistency with the results observed in the qIT screen (Table S4†). Compounds A01 and B01 were confirmed as hits against fRab27A-C188 and fRab27A-C123, respectively (Fig. 2A). The site of modification for each fragment was confirmed by tryptic digest and peptide mass fingerprinting (Fig. S5†). Hit fragment A01 preferentially reacted with fRab27A-C188, whereas fragment B01 was found to react with both fRab27A-C123 and fRab27A-C188 with significant rate enhancement over GSH (Fig. 2A).

**Fig. 3:**
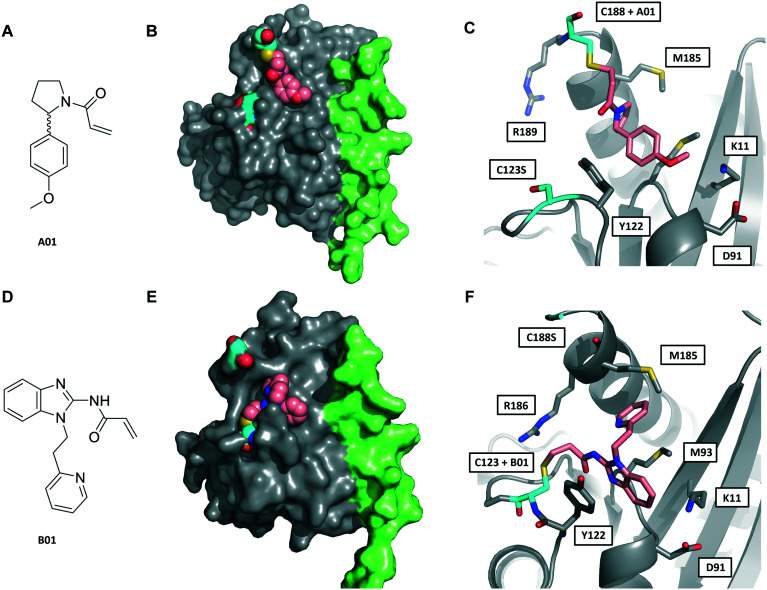
Crystal structures of fRab27A mono-cysteine mutants with ligands A01 and B01. A) A01 chemical structure. B) Crystal structure of fRab27A-C188, Rab27A (grey) fused with Slp2-SHD1 (green), with Cys188 (cyan) conjugated with A01 (pink). Flanking C123S is also highlighted in cyan. C) Atomistic view of the Rab27A SF4 pocket flanked by Cys123 mutated to serine (cyan) and Cys188 (cyan) bound to A01 (pink). Compound density map for the cys-A01 adduct is shown in Fig. S7A.† D) B01 chemical structure. E) Crystal structure of fRab27A-C123, Rab27A (grey) fused with Slp2-SHD1 (green), with Cys123 (cyan) conjugated with B01 (pink). Flanking C188S is also highlighted in cyan. F) Atomistic view of Rab27A SF4 pocket flanked by Cys188 mutated to serine (cyan) and Cys123 (cyan) bound to B01 (pink). Compound density map for the cys-B01 adduct is shown in Fig. S7B.†

We next sought to determine the binding mode of these fragments in complex with Rab27A. Hit fragments A01 and B01 were resynthesised and characterised as described in the ESI† (Schemes S1 and S2). Enhanced labelling rates for fRab27A constructs, as well as corresponding non-fusion Rab27A constructs (nRab27A), were confirmed in the qIT assay and labelling kinetics (*k*_inact_/*K*_I_) evaluated (Fig. S6†). Subsequently, fRab27A-C188 was labelled with A01 and fRab27A-C123 with B01, and the conjugates purified by gel filtration and crystallised using established conditions (see ESI†); structures were refined to 2.23 and 2.32 Å resolution, respectively (Fig. 3 and S7, Tables S2 and S3,† PDB codes: 7OPQ and 7OPR).

Although A01 contains an undefined stereocentre at the benzylic carbon (Fig. 3A), only the (*S*)-enantiomer of A01 was observed in the SF4 pocket of fRab27A-C188 (Fig. 3B and C, see Fig. S7A† for the density map). This may reflect either preferential reactivity of this enantiomer with fRab27A-C188, or alternatively preferential crystallisation of this protein–ligand complex. The phenyl ring of A01 formed an edge-on π-stacking interaction with Y122, which is known to be important for Rab27A-effector interaction, along with hydrophobic interactions with M93 and M185 (Fig. 3C). Residues K11 and D91 were located in close proximity to the methyl ether moiety, suggesting a route to build further polar contacts in analogues of A01. The structure of B01 bound to fRab27A-C123 demonstrated similar hydrophobic interactions with M93 and M185 (Fig. 3D–F, see Fig. S7B† for the density map). However, in contrast to the fRab27A-C188-A01 structures, in which the SF4 pocket is largely unperturbed by C188 modification, and the Rab27A-Slp2a complex (Fig. 1A), C123 modification by B01 caused the side chain of Y122 to adopt an alternate conformation, opening up an enlarged cavity in the pocket (Fig. 3E and F). Interestingly, molecular dynamic simulations on Rab27A (PDB: 3 BC1, chain A) suggested that the Y122 sidechain has three main rotamers with the rotamer trapped by the fRab27A-C123-B01 complex as the preferred conformation (Fig. S8A†). Y122 appears to be the residue with the highest backbone flexibility within its loop (Fig. S8B and C†) and the enhanced conformational dynamics of this region correlate with the ability of the sidechain to adopt multiple rotameric conformations. The B01 fragment was also positioned with potential fragment growth vectors to form polar interactions with K11 and D91.

## Conclusion

As noted above, Rab27A is emerging as a key regulator of metastatic behaviour in various cancers,^8–12^ and Rab27A inhibition should carry a low risk of mechanism-based toxicity in adult tissues.^14^ Like many other GTPases, Rab27A is a highly challenging target for small molecule inhibitors; however, the SF4 pocket is required for selective Rab27A-effector interactions and may afford a route to target this key PPI and disrupt function. The novel fusion construct presented here provides a new tool for structure-guided drug discovery efforts against Rab27A. Despite substantial effort, our attempts to identify non-covalent ligands of the Rab27A SF4 pocket were unsuccessful, and furthermore a range of biophysical techniques could not validate two putative reported ligands for this pocket.^15,16^ Notably, both series of proposed inhibitors contain structural motifs commonly associated with pan-assay interference substances (PAINS) that can lead to false-positive readings in many assay formats (Fig. S3†),^28^ and indeed subsequent studies have shown that at least one of these compound series has strong non-specific off-target cytotoxicity.^29^ We suggest that great caution should be employed in interpreting results from these molecules in studies of Rab27A function until target binding has been robustly demonstrated. Covalent ligands have seen a resurgence in recent years, including for targeting historically ‘undruggable’ GTPases, for example through the KRas-G12C activating mutation.^30–33^ The two unique endogenous cysteines in proximity to the SF4 pocket in Rab27A suggest a promising route to targeting through selective covalent modification, a hypothesis supported by identification of A01 and B01 fragments that react preferentially with these cysteines. Furthermore, our novel fRab27A construct has allowed determination of the first Rab27A-ligand crystal structures. These structures provide striking insights into fragment binding modes and establish key vectors for future elaboration. In summary, we present here an integrated platform for future covalent drug discovery against Rab27A, which will be a key stepping stone to validation of this important but highly challenging target.

## Author contributions

Conceptualisation, supervision and funding acquisition: EWT, EC, JCN. Investigation, methodology: MJ, TL-H, CLS, SH. Data curation, formal analysis, supervision: MJ, TL-H, MT, EDV, RP, DB, IPD, MSH. Resources, formal analysis, supervision: GBC, DJM, RMM. Visualisation: MJ, TL-H, MT, EDV. Writing – original draft, review and editing: MJ, TL-H, EDV, MT, DB. Writing – review and editing: EWT, EC.

## Conflicts of interest

EWT is a director and shareholder of Myricx Pharma Ltd; GC, DM and AA are directors and shareholders of OneFour Discovery Ltd; all other authors declare no conflict of interest.

## Supplementary Material

MD-013-D1MD00225B-s001

MD-013-D1MD00225B-s002
