# Supplementary material for: Identification of the first structurally validated covalent ligands of the small GTPase RAB27A
Source: RSC Med Chem. 2021 Dec 16;13(2):150–5. doi: 10.1039/d1md00225b (PMC8864489; doi:10.1039/d1md00225b)
Supplement: MD-013-D1MD00225B-s002 [file MD-013-D1MD00225B-s002.pdf]

Electronic Supplementary Material (ESI) for RSC Medicinal Chemistry.
This journal is © The Royal Society of Chemistry 2021

SUPPORTING INFORMATION

Identification of the first structurally validated covalent ligands of the small GTPase RAB27A

Mostafa Jamshidiha,[a,b]# Thomas Lanyon-Hogg,[a,b]# Charlotte L. Sutherell,[b] Gregory B. Craven,[a] Montse Tersa,[a] Elena De Vita,[b] Delia Brustur,[b] Inmaculada Pérez-Dorado,[a] Sarah Hassan,[b] Rita Petracca,[b] Rhodri M. Morgan,[a] Máximo Sanz-Hernández,[a] Jim C. Norman,[c] Alan Armstrong,[b] David J. Mann,[a] Ernesto Cota,*[a] and Edward W. Tate*[b]

[a] Dr M. Jamshidiha, Dr M. Tersa, Dr I. Perez-Dorado, Dr G. B. Craven, Dr R. M. Morgan, Dr M. Sanz-Hernández, Dr D. J. Mann, Dr E. Cota
Department of Life Sciences
Imperial College London
London, SW7 2AZ, UK
Email: e.cota@imperial.ac.uk

[b] Dr T. Lanyon-Hogg, Dr C. L. Sutherell, Dr E. De Vita, D. Brustur, S. Hassan, Dr R. Petracca, Prof A. Armstrong, Prof E. W. Tate
Department of Chemistry
Imperial College London
London, W12 0BZ, UK
Email: e.tate@imperial.ac.uk

[c] Prof J. C. Norman
Beatson Institute for Cancer Research
Garscube Estate
Glasgow G61 1BD, UK

# These authors contributed equally

Keywords: Rab27A, Protein-Protein interactions, Ligand screening, Covalent inhibitors, X-ray crystallography,

SUPPORTING INFORMATION 1

Supplementary Figures 3

Figure S1 3

Figure S2 4

Figure S3 5

Figure S4 14

Figure S5 15

Figure S6 16

Figure S7 17

Figure S8 18

Supplementary Tables 19

Table S1 19

Table S2 20

Table S3 21

Table S4 22

Materials and Methods 23

Protein expression and purification 23

Protein labelling and purification 23

Protein crystallisation 24

Molecular dynamics simulations 24

qIT assay for screening and hit validation 24

qIT assay for k_{inact}/K_I determination 25

Peptide mass fingerprint analysis 25

Chemical Synthesis 26

Abbreviations 26

General Information 26

Scheme S1. Synthesis of hit fragment A01 26

Scheme S2. Synthesis of hit fragment B01 29

NMR spectra for A01 and B01 32

Supplementary References 37

Supplementary Figures

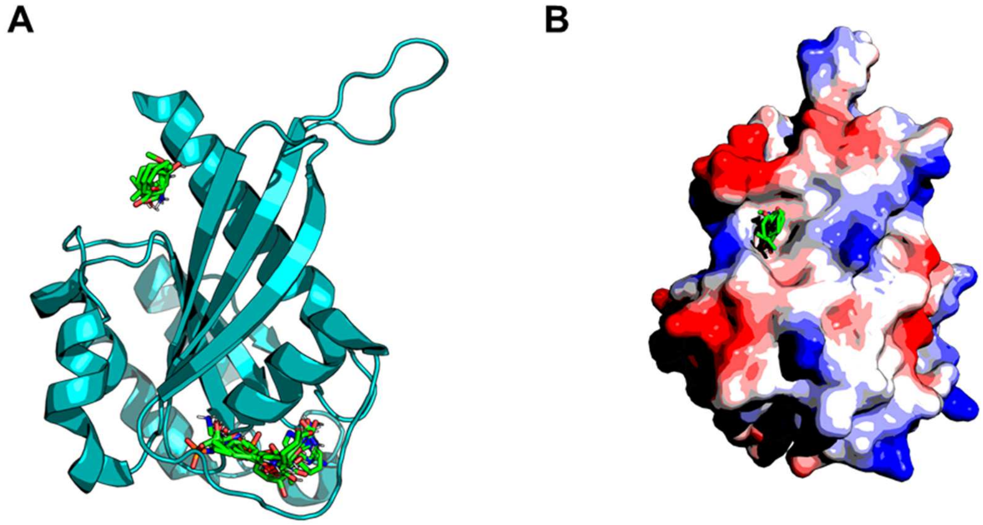

Figure S1. Evaluating Rab27A (PDB: 3BC1, chain A) hotspots using FTMap server.[1] This server identifies binding pockets within a protein surface by evaluating binding energy of molecules with different physicochemical properties. A) and B) demonstrate that organic molecule clusters predominantly occupy the nucleotide binding site and the WF pocket.

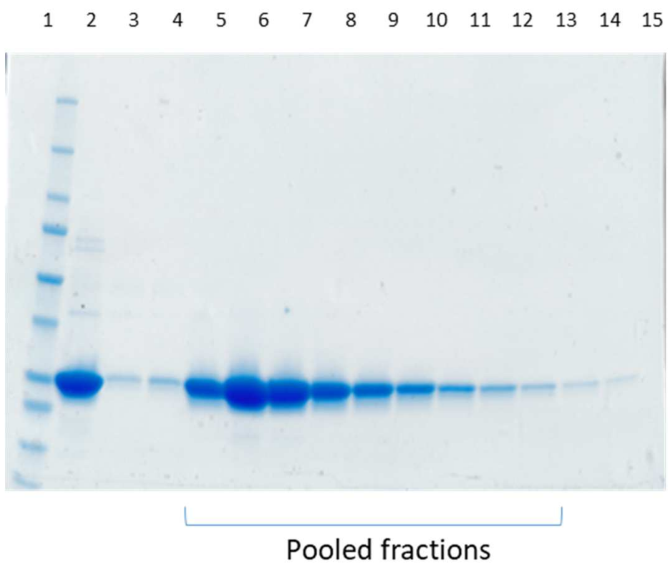

Figure S2. Last purification step for fRab27A-C188. Size-exclusion chromatography (SEC) fractions A10 to B8 were pooled to obtain pure recombinant protein. Gel lanes: 1. ladder; 2. SEC input; SEC fractions: 3. A8; 4. A9; 5. A10; 6. A11; 7. B1; 8. B3; 9. B4; 10. B5; 11. B6; 12. B7; 13. B8; 14. B9; 15. B10.

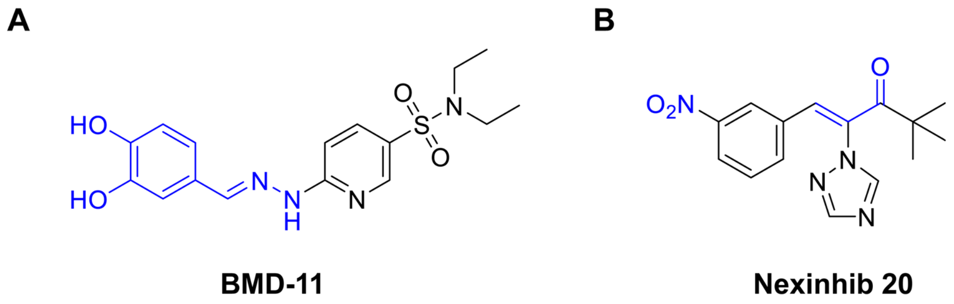

Figure S3. Structure of reported Rab27A non-covalent inhibitors: BMD-11[2] and Nexinhib 20[3]. Motifs commonly associated with PAINS are highlighted in blue, such as catechols and vinyl ketones.[4]

```
RAB27A        ------------------------------------------------------------  0
RAB27B        ------------------------------------------------------------  0

RAB24         ------------------------------------------------------------  0
RAB6C         ------------------------------------------------------------  0
RAB6D         ------------------------------------------------------------  0
RAB6A         ------------------------------------------------------------  0
RAB6B         ------------------------------------------------------------  0
RAB17         -----------------------------------------MAQ----------------  3
RAB21         -----------------------------------------MAA----------------  3
RAB5B         ------------------------------------------MT----------------  2
RAB5A         ------------------------------------------MA----------------  2
RAB5C         -----------------------------------------MAG----------------  3
RAB22A        ------------------------------------------------------------  0
RAB22B(31)    ------------------------------------------------------------  0
RAB20         ------------------------------------------------------------  0
RAB28         ------------------------------------------------------------  0
RAB29         ------------------------------------------------------------  0
RAB32         -------------------------------------------MAGGGAGDPG-----LG  12
RAB38         ------------------------------------------------------------  0
RAB34         ------------------------------------------------------------  0
RAB36         MVIAGASWMLGRAAA--SPTQTPPTTSTIRVARRSRVALVAMVIAAAGSGGPGRAEPQLS  58
RAB42         ------------------------------------------------------------  0
RAB39A        ------------------------------------------------------------  0
RAB39B        ------------------------------------------------------------  0
RAB7B         ------------------------------------------------------------  0
RAB7A         ------------------------------------------------------------  0
RAB9A         ------------------------------------------------------------  0
RAB9B         ------------------------------------------------------------  0
RAB23         ------------------------------------------------------------  0
RAB40C        ------------------------------------------------------------  0
RAB40B        ------------------------------------------------------------  0
RAB40A        ------------------------------------------------------------  0
RAB40AL       ------------------------------------------------------------  0
RAB33A        -----------------------------------------MAQPILGHGSLQPA----S  15
RAB33B        -----------------------------------------MAEEMES--SLE-A----S  12
RAB30         ------------------------------------------------------------  0
RAB19         ------------------------------------------------------------  0
RAB43         ------------------------------------------------------------  0
RAB18         ------------------------------------------------------------  0
RAB2A         ------------------------------------------------------------  0
RAB2B         ------------------------------------------------------------  0
RAB14         ------------------------------------------------------------  0
RAB4A         ------------------------------------------------------------  0
RAB4B         ------------------------------------------------------------  0
RAB25         ------------------------------------------------------------  0
RAB11A        ------------------------------------------------------------  0
RAB11B        ------------------------------------------------------------  0
RAB12         ------------------------------MDPG-----AALQRRAGGGGGLGAGSPALS  25
RAB26         ---------MSRKKTPKSKGASTPAASTLPTANG-----ARPARS--GTALSGPDAPPNG  44
RAB37         ----------------------------MTGTPG-----AVATRD--GE------APERS  19
RAB3D         -----------------------------------------------------------M  1
RAB3B         -----------------------------------------------------------M  1
RAB3A         -----------------------------------------------------------M  1
RAB3C         ---------------------------------------------------MRHEAPMQM  9
RAB15         ------------------------------------------------------------  0
RAB13         ------------------------------------------------------------  0
RAB10         ------------------------------------------------------------  0
RAB8A         ------------------------------------------------------------  0
RAB8B         ------------------------------------------------------------  0
RAB35         ------------------------------------------------------------  0
RAB1A         ------------------------------------------------------------  0
RAB1B         ------------------------------------------------------------  0
```

```
RAB27A          --------------------------------------------------------MSDG  4
RAB27B          --------------------------------------------------------MTDG  4

RAB24           ----------------------------------------------------------MS  2
RAB6C           ----------------------------------------------------MSAGGDFG  8
RAB6D           ----------------------------------------------------MSAGGDFG  8
RAB6A           ----------------------------------------------------MSTGGDFG  8
RAB6B           ----------------------------------------------------MSAGGDFG  8
RAB17           -----------------------------------------------AHR--TPQPRAAP  14
RAB21           -----------------------------------------------AGG--GGGGAAAA  14
RAB5B           -----------------------------------------------SRSTARPNGQPQA  15
RAB5A           -----------------------------------------------SRGATRPNGPNTG  15
RAB5C           -----------------------------------------------RGGAARPNGPAAG  16
RAB22A          ------------------------------------------------------------  0
RAB22B(31)      ------------------------------------------------------------  0
RAB20           ------------------------------------------------------------  0
RAB28           -----------------------------------------------------MSDSEEE  7
RAB29           ----------------------------------------------------------MG  2
RAB32           ----------------------------------------------------AAAAPAPE  20
RAB38           --------------------------------------------------------MQAP  4
RAB34           -------------MNILAPVRRDRVLAELPQCLRKEAALHGHKDFHPRVTCACQEHRTGT  47
RAB36           QPSLDCGRMRSSLTPLGPPVSRDRVIASFPKWYTPEACLQLREHFHGQVSAACQRRNTGT  118
RAB42           --------------------------------------------------------MEAE  4
RAB39A          ---------------------------------------------------------MET  3
RAB39B          ---------------------------------------------------------MEA  3
RAB7B           ---------------------------------------------------------MNP  3
RAB7A           ---------------------------------------------------------MTS  3
RAB9A           ----------------------------------------------------------MA  2
RAB9B           ----------------------------------------------------------MS  2
RAB23           --------------------------------------------------------MLEE  4
RAB40C          -------MG--------------------------------------------SQGSPVK  9
RAB40B          -------MS--------------------------------------------ALGSPVR  9
RAB40A          -------MS--------------------------------------------APGSPDQ  9
RAB40AL         -------MS--------------------------------------------APGSPDQ  9
RAB33A          AAGLASLEL--------------------------------------------DSSLDQY  31
RAB33B          FSSSGAVSG--------------------------------------------ASGFLPP  28
RAB30           --------------------------------------------------------MSME  4
RAB19           ----MHFSS--------------------------------------------SARAADE  12
RAB43           ---MAGPGP--------------------------------------------GPGDPDE  13
RAB18           ---------------------------------------------------------MDE  3
RAB2A           -----------------------------------------------------------M  1
RAB2B           -----------------------------------------------------------M  1
RAB14           ------------------------------------------------------MATAPY  6
RAB4A           --------M--------------------------------------------SQTAMSE  8
RAB4B           ---------------------------------------------------------MAE  3
RAB25           -----------------------------------------------------MGNGTEE  7
RAB11A          ------------------------------------------------------MGTRDD  6
RAB11B          ------------------------------------------------------MGTRDD  6
RAB12           --GGQGR----------------------------------------------RRKQPPR  37
RAB26           --PLQPGRP--------------------------------------------SLGGGVD  58
RAB37           --------P-----------------------------------------------PCSP  24
RAB3D           ASAGDTQAG--------------------------------------------PRDAADQ  17
RAB3B           ASVTDGKTG--------------------------------------------VKDASDQ  17
RAB3A           ASATDSRYG--------------------------------------------QKESSDQ  17
RAB3C           ASAQDARYG--------------------------------------------QKDSSDQ  25
RAB15           ---------------------------------------------------------MAK  3
RAB13           ---------------------------------------------------------MAK  3
RAB10           --------------------------------------------------------MAKK  4
RAB8A           ---------------------------------------------------------MAK  3
RAB8B           ---------------------------------------------------------MAK  3
RAB35           ---------------------------------------------------------MAR  3
RAB1A           ------------------------------------------------------MSSMNP  6
RAB1B           ---------------------------------------------------------MNP  3
```

```
RAB27A          DYDYLIKFLALGDSGVGKTSVLYQYTDGKFN-S----KFITTVGIDFREKRVVYRASGPD  59
RAB27B          DYDYLIKLLALGDSGVGKTTFLYRYTDNKFN-P----KFITTVGIDFREKRVVYNAQGPN  59

RAB24           GQRVDVKVVMLGKEYVGKTSLVERYVHDRFLVG----PYQNTIGAAFVAKVMCV------  52
RAB6C           NPLRKFKLVFLGEQSVAKTSLITRFRYDSFD-N----TYQAIIGIDFLSKTMYL------  57
RAB6D           NPLRKFKLVFLGEQSVAKTSLITRFRYDSFD-N----TYQAIIGIDFLSKTMYL------  57
RAB6A           NPLRKFKLVFLGEQSVGKTSLITRFMYDSFD-N----TYQATIGIDFLSKTMYL------  57
RAB6B           NPLRKFKLVFLGEQSVGKTSLITRFMYDSFD-N----TYQATIGIDFLSKTMYL------  57
RAB17           SQPRVFKLVLLGSGSVGKSSLALRYVKNDFK-S-----ILPTVGCAFFTKVVDV------  62
RAB21           GRAYSFKVVLLGEGCVGKTSLVLRYCENKFN-D----KHITTLQASFLTKKLNI------  63
RAB5B           SKICQFKLVLLGESAVGKSSLVLRFVKGQFH-E----YQESTIGAAFLTQSVCL------  64
RAB5A           NKICQFKLVLLGESAVGKSSLVLRFVKGQFH-E----FQESTIGAAFLTQTVCL------  64
RAB5C           NKICQFKLVLLGESAVGKSSLVLRFVKGQFH-E----YQESTIGAAFLTQTVCL------  65
RAB22A          MALRELKVCLLGDTGVGKSSIVWRFVEDSFD-P----NINPTIGASFMTKTVQY------  49
RAB22B(31)      MAIRELKVCLLGDTGVGKSSIVCRFVQDHFD-H----NISPTIGASFMTKTVPC------  49
RAB20           MRKPDSKIVLLGDMNVGKTSLLQRYMERRFP------DTVSTVGGAFYLKQWRS------  48
RAB28           SQDRQLKIVVLGDGASGKTSLTTCFAQETFG-K----QYKQTIGLDFFLRRITL------  56
RAB29           SRDHLFKVLVVGDAAVGKTSLVQRYSQDSFS-K----HYKSTVGVDFALKVLQW------  51
RAB32           TREHLFKVLVIGELGVGKTSIIKRYVHQLFS-Q----HYRATIGVDFALKVLNW------  69
RAB38           HKEHLYKLLVIGDLGVGKTSIIKRYVHQNFS-S----HYRATIGVDFALKVLHW------  53
RAB34           VGFKISKVIVVGDLSVGKTCLINRFCKDTFD-K----NYKATIGVDFEMERFEV------  96
RAB36           VGLKLSKVVVVGDLYVGKTSLIHRFCKNVFD-R----DYKATIGVDFEIERFEI------  167
RAB42           GCRYQFRVALLGDAAVGKTSLLRSYVAGAPGAPEPEPEPEPTVGAECYRRALQL------  58
RAB39A          IWIYQFRLIVIGDSTVGKSCLLHRFTQGRFPG-LRSPACDPTVGVDFFSRLLEI------  56
RAB39B          IWLYQFRLIVIGDSTVGKSCLIRRFTEGRFAQ-V----SDPTVGVDFFSRLVEI------  52
RAB7B           RKKVDLKLIIVGAIGVGKTSLLHQYVHKTFY-E----EYQTTLGASILSKIIIL------  52
RAB7A           RKKVLLKVIILGDSGVGKTSLMNQYVNKKFS-N----QYKATIGADFLTKEVMV------  52
RAB9A           GKSSLFKVILLGDGGVGKSSLMNRYVTNKFD-T----QLFHTIGVEFLNKDLEV------  51
RAB9B           GKSLLLKVILLGDGGVGKSSLMNRYVTNKFD-S----QAFHTIGVEFLNRDLEV------  51
RAB23           DMEVAIKMVVVGNGAVGKSSMIQRYCKGIFT-K----DYKKTIGVDFLERQIQV------  53
RAB40C          SYDYLLKFLLVGDSDVGKGEILESLQDGAAE-S----PYAYSNGIDYKTTTILL------  58
RAB40B          AYDFLLKFLLVGDSDVGKGEILASLQDGAAE-S----PYGHPAGIDYKTTTILL------  58
RAB40A          AYDFLLKFLLVGDRDVGKSEILESLQDGAAE-S----PYSHLGGIDYKTTTILL------  58
RAB40AL         AYDFLLKFLLVGDRDVGKSEILESLQDGTAE-S----PYSHLGGIDYKTTTILL------  58
RAB33A          VQIRIFKIIVIGDSNVGKTCLTFRFCGGTFP-D----KTEATIGVDFREKTVEI------  80
RAB33B          ARSRIFKIIVIGDSNVGKTCLTYRFCAGRFP-D----RTEATIGVDFRERAVEI------  77
RAB30           DYDFLFKIVLIGNAGVGKTCLVRRFTQGLFP-P----GQGATIGVDFMIKTVEI------  53
RAB19           NFDYLFKIILIGDSNVGKTCVVQHFKSGVYT-E----TQQNTIGVDFTVRSLDI------  61
RAB43           QYDFLFKLVLVGDASVGKTCVVQRFKTGAFS-E----RQGSTIGVDFTMKTLEI------  62
RAB18           DVLTTLKILIIGESGVGKSSLLLRFTDDTFD-P----ELAATIGVDFKVKTISV------  52
RAB2A           AYAYLFKYIIIGDTGVGKSCLLLQFTDKRFQ-P----VHDLTIGVEFGARMITI------  50
RAB2B           TYAYLFKYIIIGDTGVGKSCLLLQFTDKRFQ-P----VHDLTIGVEFGARMVNI------  50
RAB14           NYSYIFKYIIIGDMGVGKSCLLHQFTEKKFM-A----DCPHTIGVEFGTRIIEV------  55
RAB4A           TYDFLFKFLVIGNAGTGKSCLLHQFIEKKFK-D----DSNHTIGVEFGSKIINV------  57
RAB4B           TYDFLFKFLVIGSAGTGKSCLLHQFIENKFK-Q----DSNHTIGVEFGSRVVNV------  52
RAB25           DYNFVFKVVLIGESGVGKTNLLSRFTRNEFS-H----DSRTTIGVEFSTRTVML------  56
RAB11A          EYDYLFKVVLIGDSGVGKSNLLSRFTRNEFN-L----ESKSTIGVEFATRSIQV------  55
RAB11B          EYDYLFKVVLIGDSGVGKSNLLSRFTRNEFN-L----ESKSTIGVEFATRSIQV------  55
RAB12           PADFKLQVIIIGSRGVGKTSLMERFTDDTFC-E----ACKSTVGVDFKIKTVEL------  86
RAB26           FYDVAFKVMLVGDSGVGKTCLLVRFKDGAFLAG----TFISTVGIDFRNKVLDV------  108
RAB37           SYDLTGKVMLLGDTGVGKTCFLIQFKDGAFLSG----TFIATVGIDFRNKVVTV------  74
RAB3D           NFDYMFKLLLIGNSSVGKTSFLFRYADDSFT-P----AFVSTVGIDFKVKTVYR------  66
RAB3B           NFDYMFKLLIIGNSSVGKTSFLFRYADDTFT-P----AFVSTVGIDFKVKTVYR------  66
RAB3A           NFDYMFKILIIGNSSVGKTSFLFRYADDSFT-P----AFVSTVGIDFKVKTIYR------  66
RAB3C           NFDYMFKLLIIGNSSVGKTSFLFRYADDSFT-S----AFVSTVGIDFKVKTVFK------  74
RAB15           QYDVLFRLLLIGDSGVGKTCLLCRFTDNEFH-S----SHISTIGVDFKMKTIEV------  52
RAB13           AYDHLFKLLLIGDSGVGKTCLIIRFAEDNFN-N----TYISTIGIDFKIRTVDI------  52
RAB10           TYDLLFKLLLIGDSGVGKTCVLFRFSDDAFN-T----TFISTIGIDFKIKTVEL------  53
RAB8A           TYDYLFKLLLIGDSGVGKTCVLFRFSEDAFN-S----TFISTIGIDFKIRTIEL------  52
RAB8B           TYDYLFKLLLIGDSGVGKTCLLFRFSEDAFN-T----TFISTIGIDFKIRTIEL------  52
RAB35           DYDHLFKLLIIGDSGVGKSSLLLRFADNTFS-G----SYITTIGVDFKIRTVEI------  52
RAB1A           EYDYLFKLLLIGDSGVGKSCLLLRFADDTYT-E----SYISTIGVDFKIRTIEL------  55
RAB1B           EYDYLFKLLLIGDSGVGKSCLLLRFADDTYT-E----SYISTIGVDFKIRTIEL------  52
```

```
RAB27A          GATGRGQRIHLQLWDTAGQERFR-SLTTAFFRDAMGFLLLFDLTNEQSFLNVRNWISQLQ  118
RAB27B          GSSGKAFKVHLQLWDTAGQERFR-SLTTAFFRDAMGFLLMFDLTSQQSFLNVRNWMSQLQ  118

RAB24           ----GDRTVTLGIWDTAGSERYE-AMSRIYYRGAKAAIVCYDLTDSSSFERAKFWVKELR  107
RAB6C           ----EDGTIGLRLWDTAGQERLR-SLIPRYIRDSAAAVVVYDITNVNSFQQTTKWIDDVR  112
RAB6D           ----EDGTIGLRLWDTAGQERLR-SLIPRYIRDSAAAVVVYDITNVNSFQQTTKWIDDVR  112
RAB6A           ----EDRTVRLQLWDTAGQERFR-SLIPSYIRDSTVAVVVYDITNVNSFQQTTKWIDDVR  112
RAB6B           ----EDRTVRLQLWDTAGQERFR-SLIPSYIRDSTVAVVVYDITNLNSFQQTSKWIDDVR  112
RAB17           ----GATSLKLEIWDTAGQEKYH-SVCHLYFRGANAALLVYDITRKDSFLKAQQWLKDLE  117
RAB21           ----GGKRVNLAIWDTAGQERFH-ALGPIYYRDSNGAILVYDITDEDSFQKVKNWVKELR  118
RAB5B           ----DDTTVKFEIWDTAGQERYH-SLAPMYYRGAQAAIVVYDITNQETFARAKTWVKELQ  119
RAB5A           ----DDTTVKFEIWDTAGQERYH-SLAPMYYRGAQAAIVVYDITNEESFARAKNWVKELQ  119
RAB5C           ----DDTTVKFEIWDTAGQERYH-SLAPMYYRGAQAAIVVYDITNTDTFARAKNWVKELQ  120
RAB22A          ----QNELHKFLIWDTAGQERFR-ALAPMYYRGSAAAIIVYDITKEETFSTLKNWVKELR  104
RAB22B(31)      ----GNELHKFLIWDTAGQERFH-SLAPMYYRGSAAAVIVYDITKQDSFYTLKKWVKELK  104
RAB20           --------YNISIWDTAGREQFH-GLGSMYCRGAAAIILTYDVNHRQSLVELEDRFLGLT  99
RAB28           ---PGNLNVTLQIWDIGGQTIGG-KMLDKYIYGAQGVLLVYDITNYQSFENLEDWYTVVK  112
RAB29           ---SDYEIVRLQLWDIAGQERFT-SMTRLYYRDASACVIMFDVTNATTFSNSQRWKQDLD  107
RAB32           ---DSRTLVRLQLWDIAGQERFG-NMTRVYYKEAVGAFVVFDISRSSTFEAVLKWKSDLD  125
RAB38           ---DPETVVRLQLWDIAGQERFG-NMTRVYYREAMGAFIVFDVTRPATFEAVAKWKNDLD  109
RAB34           ----LGIPFSLQLWDTAGQERFK-CIASTYYRGAQAIIIVFNLNDVASLEHTKQWLADAL  151
RAB36           ----AGIPYSLQIWDTAGQEKFK-CIASAYYRGAQVIITAFDLTDVQTLEHTRQWLEDAL  222
RAB42           ---RAGPRVKLQLWDTAGHERFR-CITRSFYRNVVGVLLVFDVTNRKSFEHIQDWHQEVM  114
RAB39A          ---EPGKRIKLQLWDTAGQERFR-SITRSYYRNSVGGFLVFDITNRRSFEHVKDWLEEAK  112
RAB39B          ---EPGKRIKLQIWDTAGQERFR-SITRAYYRNSVGGLLLFDITNRRSFQNVHEWLEETK  108
RAB7B           ----GDTTLKLQIWDTGGQERFR-SMVSTFYKGSDGCILAFDVTDLESFEALDIWRGDVL  107
RAB7A           ----DDRLVTMQIWDTAGQERFQ-SLGVAFYRGADCCVLVFDVTAPNTFKTLDSWRDEFL  107
RAB9A           ----DGHFVTMQIWDTAGQERFR-SLRTPFYRGSDCCLLTFSVDDSQSFQNLSNWKKEFI  106
RAB9B           ----DGRFVTLQIWDTAGQERFK-SLRTPFYRGADCCLLTFSVDDRQSFENLGNWQKEFI  106
RAB23           ----NDEDVRLMLWDTAGQEEFD-AITKAYYRGAQACVLVFSTTDRESFEAVSSWREKVV  108
RAB40C          ----DGRRVKLELWDTSGQGRFC-TIFRSYSRGAQGILLVYDITNRWSFDGIDRWIKEID  113
RAB40B          ----DGRRVKLQLWDTSGQGRFC-TIFRSYSRGAQGVILVYDIANRWSFDGIDRWIKEID  113
RAB40A          ----DGQRVKLKLWDTSGQGRFC-TIFRSYSRGAQGVILVYDIANRWSFEGMDRWIKKIE  113
RAB40AL         ----DGQRVKLKLWDTSGQGRFC-TIFRSYSRGAQGVILVYDIANRWSFEGMDRWIKKIE  113
RAB33A          ----EGEKIKVQVWDTAGQERFRKSMVEHYYRNVHAVVFVYDVTKMTSFTNLKMWIQECN  136
RAB33B          ----DGERIKIQLWDTAGQERFRKSMVQHYYRNVHAVVFVYDMTNMASFHSLPSWIEECK  133
RAB30           ----NGEKVKLQIWDTAGQERFR-SITQSYYRSANALILTYDITCEESFRCLPEWLREIE  108
RAB19           ----DGKKVKMQVWDTAGQERFR-TITQSYYRSAHAAIIAYDLTRRSTFESIPHWIHEIE  116
RAB43           ----QGKRVKLQIWDTAGQERFR-TITQSYYRSANGAILAYDITKRSSFLSVPHWIEDVR  117
RAB18           ----DGNKAKLAIWDTAGQERFR-TLTPSYYRGAQGVILVYDVTRRDTFVKLDNWLNELE  107
RAB2A           ----DGKQIKLQIWDTAGQESFR-SITRSYYRGAAGALLVYDITRRDTFNHLTTWLEDAR  105
RAB2B           ----DGKQIKLQIWDTAGQESFR-SITRSYYRGAAGALLVYDITRRETFNHLTSWLEDAR  105
RAB14           ----SGQKIKLQIWDTAGQERFR-AVTRSYYRGAAGALMVYDITRRSTYNHLSSWLTDAR  110
RAB4A           ----GGKYVKLQIWDTAGQERFR-SVTRSYYRGAAGALLVYDITSRETYNALTNWLTDAR  112
RAB4B           ----GGKTVKLQIWDTAGQERFR-SVTRSYYRGAAGALLVYDITSRETYNSLAAWLTDAR  107
RAB25           ----GTAAVKAQIWDTAGLERYR-AITSAYYRGAVGALLVFDLTKHQTYAVVERWLKELY  111
RAB11A          ----DGKTIKAQIWDTAGQERYR-AITSAYYRGAVGALLVYDIAKHLTYENVERWLKELR  110
RAB11B          ----DGKTIKAQIWDTAGQERYR-AITSAYYRGAVGALLVYDIAKHLTYENVERWLKELR  110
RAB12           ----RGKKIRLQIWDTAGQERFN-SITSAYYRSAKGIILVYDITKKETFDDLPKWMKMID  141
RAB26           ----DGVKVKLQMWDTAGQERFR-SVTHAYYRDAHALLLLYDVTNKASFDNIQAWLTEIH  163
RAB37           ----DGVRVKLQIWDTAGQERFR-SVTHAYYRDAQALLLLYDITNKSSFDNIRAWLTEIH  129
RAB3D           ----HDKRIKLQIWDTAGQERYR-TITTAYYRGAMGFLLMYDIANQESFAAVQDWATQIK  121
RAB3B           ----HEKRVKLQIWDTAGQERYR-TITTAYYRGAMGFILMYDITNEESFNAVQDWATQIK  121
RAB3A           ----NDKRIKLQIWDTAGQERYR-TITTAYYRGAMGFILMYDITNEESFNAVQDWSTQIK  121
RAB3C           ----NEKRIKLQIWDTAGQERYR-TITTAYYRGAMGFILMYDITNEESFNAVQDWSTQIK  129
RAB15           ----DGIKVRIQIWDTAGQERYQ-TITKQYYRRAQGIFLVYDISSERSYQHIMKWVSDVD  107
RAB13           ----EGKKIKLQVWDTAGQERFK-TITTAYYRGAMGIILVYDITDEKSFENIQNWMKSIK  107
RAB10           ----QGKKIKLQIWDTAGQERFH-TITTSYYRGAMGIMLVYDITNGKSFENISKWLRNID  108
RAB8A           ----DGKRIKLQIWDTAGQERFR-TITTAYYRGAMGIMLVYDITNEKSFDNIRNWIRNIE  107
RAB8B           ----DGKKIKLQIWDTAGQERFR-TITTAYYRGAMGIMLVYDITNEKSFDNIKNWIRNIE  107
RAB35           ----NGEKVKLQIWDTAGQERFR-TITSTYYRGTHGVIVVYDVTSAESFVNVKRWLHEIN  107
RAB1A           ----DGKTIKLQIWDTAGQERFR-TITSSYYRGAHGIIVVYDVTDQESFNNVKQWLQEID  110
RAB1B           ----DGKTIKLQIWDTAGQERFR-TITSSYYRGAHGIIVVYDVTDQESYANVKQWLQEID  107
```

```
RAB27A          MHAYC---ENPDIVLCGNKSDLED----QRVV-KEEE-----------------------  147
RAB27B          ANAYC---ENPDIVLIGNKADLPD----QREV-NERQ-----------------------  147

RAB24           SLEEG-----CQIYLCGTKSDLLEEDRRRRRV-DFHD-----------------------  138
RAB6C           TERGS----DVIITLVGNRTDLAD----KRQV-SVEE-----------------------  140
RAB6D           TEGGS----DVIITLVGNKTDLAD----KRQV-SIEE-----------------------  140
RAB6A           TERGS----DVIIMLVGNKTDLAD----KRQV-SIEE-----------------------  140
RAB6B           TERGS----DVIIMLVGNKTDLAD----KRQI-TIEE-----------------------  140
RAB17           EELHP---GEVLVMLVGNKTDLSQ----EREV-TFQE-----------------------  146
RAB21           KMLGN----EICLCIVGNKIDLEK----ERHV-SIQE-----------------------  146
RAB5B           RQASP----SIVIALAGNKADLAN----KRMV-EYEE-----------------------  147
RAB5A           RQASP----NIVIALSGNKADLAN----KRAV-DFQE-----------------------  147
RAB5C           RQASP----NIVIALAGNKADLAS----KRAV-EFQE-----------------------  148
RAB22A          QHGPP----NIVVAIAGNKCDLID----VREV-MERD-----------------------  132
RAB22B(31)      EHGPE----NIVMAIAGNKCDLSD----IREV-PLKD-----------------------  132
RAB20           DTASK----DCLFAIVGNKVDLTE----EGAL-AGQEKEECSPNMDAGDRVSPRAPKQVQ  150
RAB28           KVSEE-SETQPLVALVGNKIDLEH----MRTI-KPEK-----------------------  143
RAB29           SKLTLPNGEPVPCLLLANKCDLSP----WAV--SRDQ-----------------------  138
RAB32           SKVHLPNGSPIPAVLLANKCDQNK----DSSQ-SPSQ-----------------------  157
RAB38           SKLSLPNGKPVSVVLLANKCDQGK----DVLMNNGLK-----------------------  142
RAB34           KENDP---SSVLLFLVGSKKDLST----PAQY-ALMEKD---------------------  182
RAB36           RENEA---GSCFIFLVGTKKDLLS----GAAC-EQAEAD---------------------  253
RAB42           ATQGP---DKVIFLLVGHKSDLQS----TRCV-SAQE-----------------------  143
RAB39A          MYVQP---FRIVFLLVGHKCDLAS----QRQV-TREE-----------------------  141
RAB39B          VHVQP---YQIVFVLVGHKCDLDT----QRQV-TRHE-----------------------  137
RAB7B           AKIVP-MEQSYPMVLLGNKIDLA-----DRKV-PQEV-----------------------  137
RAB7A           IQASPRDPENFPFVVLGNKIDLE-----NRQV-ATKR-----------------------  138
RAB9A           YYADVKEPESFPFVILGNKIDIS-----ERQV-STEE-----------------------  137
RAB9B           YYADVKDPEHFPFVVLGNKVDKE-----DRQV-TTEE-----------------------  137
RAB23           AEVG-----DIPTVLVQNKIDLLD----DSCI-KNEE-----------------------  135
RAB40C          EHAP-----GVPRILVGNRLHLAF----KRQV-PTEQ-----------------------  140
RAB40B          EHAP-----GVPKILVGNRLHLAF----KRQV-PTEQ-----------------------  140
RAB40A          EHAP-----GVPKILVGNRLHLAF----KRQV-PREQ-----------------------  140
RAB40AL         EHAP-----GVPKILVGNRLHLAF----KRQV-PREQ-----------------------  140
RAB33A          GHAVP---PLVPKVLVGNKCDLRE----QIQV-PSNL-----------------------  165
RAB33B          QHLLA---NDIPRILVGNKCDLRS----AIQV-PTDL-----------------------  162
RAB30           QYASN----KVITVLVGNKIDLAE----RREV-SQQR-----------------------  136
RAB19           KYGAA----NVVIMLIGNKCDLWE----KRHV-LFED-----------------------  144
RAB43           KYAGS----NIVQLLIGNKSDLSE----LREV-SLAE-----------------------  145
RAB18           TYCTR---NDIVNMLVGNKIDKE-----NREV-DRNE-----------------------  135
RAB2A           QHSNS----NMVIMLIGNKSDLES----RREV-KKEE-----------------------  133
RAB2B           QHSSS----NMVIMLIGNKSDLES----RRDV-KREE-----------------------  133
RAB14           NLTNP----NTVIILIGNKADLEA----QRDV-TYEE-----------------------  138
RAB4A           MLASQ----NIVIILCGNKKDLDA----DREV-TFLE-----------------------  140
RAB4B           TLASP----NIVVILCGNKKDLDP----EREV-TFLE-----------------------  135
RAB25           DHAEA----TIVVMLVGNKSDLSQ----AREV-PTEE-----------------------  139
RAB11A          DHADS----NIVIMLVGNKSDLRH----LRAV-PTDE-----------------------  138
RAB11B          DHADS----NIVIMLVGNKSDLRH----LRAV-PTDE-----------------------  138
RAB12           KYASE----DAELLLVGNKLDCET----DREI-TRQQ-----------------------  169
RAB26           EYAQH----DVALMLLGNKVDSAH----ERVV-KRED-----------------------  191
RAB37           EYAQR----DVVIMLLGNKADMSS----ERVI-RSED-----------------------  157
RAB3D           TYSWD----NAQVILVGNKCDLED----ERVV-PAED-----------------------  149
RAB3B           TYSWD----NAQVILVGNKCDMEE----ERVV-PTEK-----------------------  149
RAB3A           TYSWD----NAQVLLVGNKCDMED----ERVV-SSER-----------------------  149
RAB3C           TYSWD----NAQVILVGNKCDMED----ERVI-STER-----------------------  157
RAB15           EYAPE----GVQKILIGNKADEEQ----KRQV-GREQ-----------------------  135
RAB13           ENASA----GVERLLLGNKCDMEA----KRKV-QKEQ-----------------------  135
RAB10           EHANE----DVERMLLGNKCDMDD----KRVV-PKGK-----------------------  136
RAB8A           EHASA----DVEKMILGNKCDVND----KRQV-SKER-----------------------  135
RAB8B           EHASS----DVERMILGNKCDMND----KRQV-SKER-----------------------  135
RAB35           QNC-D----DVCRILVGNKNDDPE----RKVV-ETED-----------------------  134
RAB1A           RYASE----NVNKLLVGNKCDLTT----KKVV-DYTT-----------------------  138
RAB1B           RYASE----NVNKLLVGNKSDLTT----KKVV-DNTT-----------------------  135
```

```
RAB27A          ---AIALAEK-Y------------GI-PYFETSAAN---GTNISQAIEMLLDLIMKRMER  187
RAB27B          ---ARELADK-Y------------GI-PYFETSAAT---GQNVEKAVETLLDLIMKRMEQ  187

RAB24           ---VQDYADN-I------------KA-QLFETSSKT---GQSVDELFQKVAEDYVSVAAF  178
RAB6C           ---GERKAKG-L------------NV-TFIETRAKA---GYNVKQLFRRVAAALPGMEST  180
RAB6D           ---GERKAKG-L------------NV-TFIETRAKA---GYNVKQLFRRVAAALPGMEST  180
RAB6A           ---GERKAKE-L------------NV-MFIETSAKA---GYNVKQLFRRVAAALPGMEST  180
RAB6B           ---GEQRAKE-L------------SV-MFIETSAKT---GYNVKQLFRRVASALPGMENV  180
RAB17           ---GKEFADS-Q------------KL-LFMETSAKL---NHQVSEVFNTVAQELLQRSDE  186
RAB21           ---AESYAES-V------------GA-KHYHTSAKQ---NKGIEELFLDLCKRMIETAQV  186
RAB5B           ---AQAYADD-N------------SL-LFMETSAKT---AMNVNDLFLAIAKKLPKSEPQ  187
RAB5A           ---AQSYADD-N------------SL-LFMETSAKT---SMNVNEIFMAIAKKLPKNEPQ  187
RAB5C           ---AQAYADD-N------------SL-LFMETSAKT---AMNVNEIFMAIAKKLPKNEPQ  188
RAB22A          ---AKDYADS-I------------HA-IFVETSAKN---AININELFIEISRRIPSTDAN  172
RAB22B(31)      ---AKEYAES-I------------GA-IVVETSAKN---AINIEELFQGISRQIPPLDPH  172
RAB20           LEDAVALYKKILKYKMLDEQDVPAAEQMCFETSAKT---GYNVDLLFETLFDLVVPMILQ  207
RAB28           ---HLRFCQE-N------------GF-SSHFVSAKT---GDSVFLCFQKVAAEILGIKLN  183
RAB29           ---IDRFSKE-N------------GFTGWTETSVKE---NKNINEAMRVLIEKMMRNSTE  179
RAB32           ---VDQFCKE-H------------GFAGWFETSAKD---NINIEEAARFLVEKILVNHQS  198
RAB38           ---MDQFCKE-H------------GFVGWFETSAKE---NINIDEASRCLVKHILANECD  183
RAB34           ---ALQVAQE-M------------KA-EYWAVSSLT---GENVREFFFRVAALTFEANVL  222
RAB36           ---AVHLARE-M------------QA-EYWSVSAKT---GENVKAFFSRVAALAFEQSVL  293
RAB42           ---AEELAAS-L------------GM-AFVETSVKN---NCNVDLAFDTLADAIQQALQQ  183
RAB39A          ---AEKLSAD-C------------GM-KYIETSAKD---ATNVEESFTILTRDIYELIKK  181
RAB39B          ---AEKLAAA-Y------------GM-KYIETSARD---AINVEKAFTDLTRDIYELVKR  177
RAB7B           ---AQGWCRE-K-------------DIPYFEVSAKN---DINVVQAFEMLASRALSRYQS  177
RAB7A           ---AQAWCYS-K------------NNIPYFETSAKE---AINVEQAFQTIARNALKQETE  179
RAB9A           ---AQAWCRD-N------------GDYPYFETSAKD---ATNVAAAFEEAVRRVLATEDR  178
RAB9B           ---AQTWCME-N------------GDYPYLETSAKD---DTNVTVAFEEAVRQVLAVEEQ  178
RAB23           ---AEALAKR-L------------KL-RFYRTSVKE---DLNVNEVFKYLAEKYLQKLKQ  175
RAB40C          ---ARAYAEK-N------------CM-TFFEVSPLC---NFNVIESFTELSRIVLMRHGM  180
RAB40B          ---AQAYAER-L------------GV-TFFEVSPLC---NFNITESFTELARIVLLRHGM  180
RAB40A          ---AQAYAER-L------------GV-TFFEVSPLC---NFNIIESFTELARIVLLRHRM  180
RAB40AL         ---AQAYAER-L------------GV-TFFEVSPLC---NFNIIESFTELARIVLLRHRL  180
RAB33A          ---ALKFADA-H------------NM-LLFETSAKDPKESQNVESIFMCLACRLKAQKSL  208
RAB33B          ---AQKFADT-H------------SM-PLFETSAKNPNDNDHVEAIFMTLAHKLKSHKPL  205
RAB30           ---AEEFSEA-Q------------DM-YYLETSAKE---SDNVEKLFLDLACRLISEARQ  176
RAB19           ---ACTLAEK-Y------------GLLAVLETSAKE---SKNIEEVFVLMAKELIARNSL  185
RAB43           ---AQSLAEH-Y------------DILCAIETSAKD---SSNVEEAFLRVATELIMRHGG  186
RAB18           ---GLKFARK-H------------SM-LFIEASAKT---CDGVQCAFEELVEKIIQTPGL  175
RAB2A           ---GEAFARE-H------------GL-IFMETSAKT---ASNVEEAFINTAKEIYEKIQE  173
RAB2B           ---GEAFARE-H------------GL-IFMETSAKT---ACNVEEAFINTAKEIYRKIQQ  173
RAB14           ---AKQFAEE-N------------GL-LFLEASAKT---GENVEDAFLEAAKKIYQNIQD  178
RAB4A           ---ASRFAQE-N------------EL-MFLETSALT---GENVEEAFVQCARKILNKIES  180
RAB4B           ---ASRFAQE-N------------EL-MFLETSALT---GENVEEAFLKCARTILNKIDS  175
RAB25           ---ARMFAEN-N------------GL-LFLETSALD---STNVELAFETVLKEIFAKVSK  179
RAB11A          ---ARAFAEK-N------------GL-SFIETSALD---STNVEAAFQTILTEIYRIVSQ  178
RAB11B          ---ARAFAEK-N------------NL-SFIETSALD---STNVEEAFKNILTEIYRIVSQ  178
RAB12           ---GEKFAQQIT------------GM-RFCEASAKD---NFNVDEIFLKLVDDILKKMPL  210
RAB26           ---GEKLAKE-Y------------GL-PFMETSAKT---GLNVDLAFTAIAKELKQRSMK  231
RAB37           ---GETLARE-Y------------GV-PFLETSAKT---GMNVELAFLAIAKELKYRAGH  197
RAB3D           ---GRRLADD-L------------GF-EFFEASAKE---NINVKQVFERLVDVICEKMNE  189
RAB3B           ---GQLLAEQ-L------------GF-DFFEASAKE---NISVRQAFERLVDAICDKMSD  189
RAB3A           ---GRQLADH-L------------GF-EFFEASAKD---NINVKQTFERLVDVICEKMSE  189
RAB3C           ---GQHLGEQ-L------------GF-EFFETSAKD---NINVKQTFERLVDIICDKMSE  197
RAB15           ---GQQLAKE-Y------------GM-DFYETSACT---NLNIKESFTRLTELVLQAHRK  175
RAB13           ---ADKLARE-H------------GI-RFFETSAKS---SMNVDEAFSSLARDILLKSGG  175
RAB10           ---GEQIARE-H------------GI-RFFETSAKA---NINIEKAFLTLAEDILRKTPV  176
RAB8A           ---GEKLALD-Y------------GI-KFMETSAKA---NINVENAFFTLARDIKAKMDK  175
RAB8B           ---GEKLAID-Y------------GI-KFLETSAKS---SANVEEAFFTLARDIMTKLNR  175
RAB35           ---AYKFAGQ-M------------GI-QLFETSAKE---NVNVEEMFNCITELVLRAKKD  174
RAB1A           ---AKEFADS-L------------GI-PFLETSAKN---ATNVEQSFMTMAAEIKKRMGP  178
RAB1B           ---AKEFADS-L------------GI-PFLETSAKN---ATNVEQAFMTMAAEIKKRMGP  175
```

```
RAB27A          CVDKSWIPEGV-VRSNGHA------------------S-TD---------QLSEEKEKGA  218
RAB27B          CVEKTQIPDTV-NGGNSGN-------------------------------LDGEKPPEKK  215

RAB24           QVMTE-----------------------------DKGV---DLS------QK-ANPYFYS  199
RAB6C           QDGSRE------------------------------DMSDIKLE------KPQEQTVSEG  204
RAB6D           QDGSRE------------------------------DMSDIKLE------KPQEQTVSEG  204
RAB6A           QDRSRE------------------------------DMIDIKLE------KPQEQPVSEG  204
RAB6B           QEKSKE------------------------------GMIDIKLD------KPQEPPASEG  204
RAB17           EGQA------------------------------LRGDAAVALN-------K-GPARQAK  208
RAB21           DERAKGNGSSQ-------------------PGTARRGVQIIDDE------PQ-AQTSGGG  220
RAB5B           NLG---G---A-------------------A-GRSRGV---DLH------EQ-SQQNKSQ  211
RAB5A           NPG---A---N-------------------S-ARGRGV---DLT------EP-TQPTRNQ  211
RAB5C           NAT---G---A-------------------P-GRNRGV---DLQ------EN-NPASRSQ  212
RAB22A          LPS---G---------------------------GKGF---KLR------RQ-PSEPKRS  192
RAB22B(31)      ENG---N---------------------------NGTI---KVE------KP-TMQASRR  192
RAB20           QRAERPSHTVD-------------------------------ISS-----HKPPKRTRSG  231
RAB28           KAEIEQ-SQRVVKA-----------------------D-IVNYNQEPMS---RTVNPPRS  215
RAB29           DIMSLSTQG----------------------------D-YINLQTK-----S----SSWS  201
RAB32           FPNE-ENDV----------------------------D-KIKLDQE-----TLRAENKSQ  223
RAB38           LMESIEPDV----------------------------V-KPHLT-------STKVASCSG  207
RAB34           AELEKSGARRI-------------------------GD-VVRINSDDSN-LYLTASKKKP  255
RAB36           QDLERQSSARL-----QV----------------GNGD-LIQMEGSPPE-TQESKRPSSL  330
RAB42           GDIKLEEGWGGVRLI----------------------H-KTQIPRS----P-SRKQHSGP  215
RAB39A          GEICIQDGWEGVKSG----------------------F-VPNTVHS----SEEAVKPRKE  214
RAB39B          GEITIQEGWEGVKSG----------------------F-VPNVVHS----SEEVVKSERR  210
RAB7B           ILE-N--HLTE----------------------------SIKL-------S-P-DQSRSR  197
RAB7A           VELYNEFPEPI----------------------------KLDK-------NDR-AKASAE  203
RAB9A           SDHLI---QTD----------------------------TVNL-------HRK-PKPSSS  199
RAB9B           LEHCM---LGH----------------------------TIDL-------NSG-SKAGSS  199
RAB23           QIAEDPELTHSSSNKIGVFNTSGGSHSGQNSGTLNGGD-VINLRPN-KQRTKKNRNPFSS  233
RAB40C          EKIWRPNRV---------------------------------------------FSLQDL  195
RAB40B          DRLWRPSKV---------------------------------------------LSLQDL  195
RAB40A          NWLGRPSKV---------------------------------------------LSLQDL  195
RAB40AL         NWLGRPSKV---------------------------------------------LSLQDL  195
RAB33A          LYRDAERQQGK----V---------------------Q-KLEF--------PQEANSKTS  234
RAB33B          MLSQPPDN-G------------------------------IIL--------KPEPKPAMT  226
RAB30           NTLVNNV--------------------------------SSPL-------PGEGKSISYL  197
RAB19           HLYGESALN-G----LPLD------------------S-SPVL-------MAQGPSEKTH  214
RAB43           PLFSEKSPD-H----IQLN------------------S-KDI-------------GEGWG  209
RAB18           WESENQNK--G----VKLS------------------H-REE--------GQG-GGACGG  201
RAB2A           GVFDINNEANGIKIGPQHA------------------A-TNATHAG----NQGGQQAGGG  210
RAB2B           GLFDVHNEANGIKIGPQQS------------------I-STSVGPSASQRNSRDIGSNSG  214
RAB14           GSLDLNAAESGVQHKPSAP------------------Q-GGRL-TS----EPQPQREGCG  214
RAB4A           GELDPERMGSGIQYGDAAL------------------R-QLRSPRR----AQAPNAQECG  217
RAB4B           GELDPERMGSGIQYGDASL------------------R-QLRQPRS----AQAVAPQPCG  212
RAB25           QRQNSIRTNAITLGSAQ---------------------------AG----QEPGPGEKRA  208
RAB11A          KQMSDRRENDMSPSNNVV---------------------PIHVPPT----TEN--KPKVQ  211
RAB11B          KQIADRAAHDESPGNNVV---------------------DISVPPT----TDGQKPNKLQ  213
RAB12           DILRNELS--NSI--LSLQ------------------P-EPEIPPE----LPP-PRPHVR  242
RAB26           APSEPRFR---------LH------------------D-YV----------KR-EGRGAS  252
RAB37           QADEPSFQ---------IR------------------D-YV----------ES-QKKRSS  218
RAB3D           SLEPSSSS-GSNGKGPAVG------------------D-AP-------------APQPSS  216
RAB3B           SLDT-DPSMLGSSKNTRLS------------------D-TP-------------PLLQQN  216
RAB3A           SLDTADPAVTGAKQGPQLS------------------D-QQ-------------VPPHQD  217
RAB3C           SLET-DPAITAAKQNTRLK------------------E-TP-------------PPPQPN  224
RAB15           ELEGLRMRASNELALAELE------------------E-EEG-KPE----GP--ANSSKT  209
RAB13           RRSGNGNKPPSTD--LK---------------------------------TCD-KKNTNK  199
RAB10           KEPNSENVDISSGGGVT---------------------------GW----KS-------K  198
RAB8A           KLEGNSPQ--GSNQGVKIT-------------------------PD----QQK-RSSFFR  203
RAB8B           KMNDSNSA--GAGGPVKIT-------------------------EN----RSK-KTSFFR  203
RAB35           NLAKQQQQ--QQNDVVKLT-------------------------KN--------SKRKKR  199
RAB1A           GATAGGAE--KSNVKIQST-------------------------PV--------KQSGGG  203
RAB1B           GAASGG-E--RPNLKIDST-------------------------PV--------KPAGGG  199
```

```
RAB27A          CGC---------------------------------------------------------  221
RAB27B          CIC---------------------------------------------------------  218

RAB24           CCHH--------------------------------------------------------  203
RAB6C           GCSCYSPMSSSTLPQKPPYSFIDCSVNIGLNLFPSLITFCNSSLLPVSWR----------  254
RAB6D           GCSCYSPMSSSTLPQKPPYSFIDCSVNIGLNLFPSLITFCNSSLLPVSWR----------  254
RAB6A           GCSC--------------------------------------------------------  208
RAB6B           GCSC--------------------------------------------------------  208
RAB17           CCAH--------------------------------------------------------  212
RAB21           CCSSG-------------------------------------------------------  225
RAB5B           CCSN--------------------------------------------------------  215
RAB5A           CCSN--------------------------------------------------------  215
RAB5C           CCSN--------------------------------------------------------  216
RAB22A          CC----------------------------------------------------------  194
RAB22B(31)      CC----------------------------------------------------------  194
RAB20           CCA---------------------------------------------------------  234
RAB28           SMCAVQ------------------------------------------------------  221
RAB29           CC----------------------------------------------------------  203
RAB32           CC----------------------------------------------------------  225
RAB38           CAKS--------------------------------------------------------  211
RAB34           TCCP--------------------------------------------------------  259
RAB36           GCC---------------------------------------------------------  333
RAB42           CQC---------------------------------------------------------  218
RAB39A          CFC---------------------------------------------------------  217
RAB39B          CLC---------------------------------------------------------  213
RAB7B           CC----------------------------------------------------------  199
RAB7A           SCSC--------------------------------------------------------  207
RAB9A           CC----------------------------------------------------------  201
RAB9B           CC----------------------------------------------------------  201
RAB23           CSIP--------------------------------------------------------  237
RAB40C          CCRAIVSCTPVHLIDKLPLPVTIKS---HLKSFSMANGMNAVMMHGRSYSLASGAGGGGS  252
RAB40B          CCRAVVSCTPVHLVDKLPLPIALRS---HLKSFSMANGLNARMMHGGSYSLTTSST---H  249
RAB40A          CCRTIVSCTPVHLVDKLPLPSTLRS---HLKSFSMAKGLNARMMRGLSYSLTTSST---H  249
RAB40AL         CCRTIVSCTPVHLVDKLPLPIALRS---HLKSFSMAKGLNARMMRGLSYSLTTSST---H  249
RAB33A          CPC---------------------------------------------------------  237
RAB33B          CWC---------------------------------------------------------  229
RAB30           TCCNFN------------------------------------------------------  203
RAB19           CTC---------------------------------------------------------  217
RAB43           CGC---------------------------------------------------------  212
RAB18           YCSVL-------------------------------------------------------  206
RAB2A           CC----------------------------------------------------------  212
RAB2B           CC----------------------------------------------------------  216
RAB14           C-----------------------------------------------------------  215
RAB4A           C-----------------------------------------------------------  218
RAB4B           C-----------------------------------------------------------  213
RAB25           CCISL-------------------------------------------------------  213
RAB11A          CCQNI-------------------------------------------------------  216
RAB11B          CCQNL-------------------------------------------------------  218
RAB12           CC----------------------------------------------------------  244
RAB26           CCRP--------------------------------------------------------  256
RAB37           CCSFM-------------------------------------------------------  223
RAB3D           CSC---------------------------------------------------------  219
RAB3B           CSC---------------------------------------------------------  219
RAB3A           CAC---------------------------------------------------------  220
RAB3C           CAC---------------------------------------------------------  227
RAB15           CWC---------------------------------------------------------  212
RAB13           CSLG--------------------------------------------------------  203
RAB10           CC----------------------------------------------------------  200
RAB8A           CVLL--------------------------------------------------------  207
RAB8B           CSLL--------------------------------------------------------  207
RAB35           CC----------------------------------------------------------  201
RAB1A           CC----------------------------------------------------------  205
RAB1B           CC----------------------------------------------------------  201
```

```
RAB27A          -----------------------------   221
RAB27B          -----------------------------   218

RAB24           -----------------------------   203
RAB6C           -----------------------------   254
RAB6D           -----------------------------   254
RAB6A           -----------------------------   208
RAB6B           -----------------------------   208
RAB17           -----------------------------   212
RAB21           -----------------------------   225
RAB5B           -----------------------------   215
RAB5A           -----------------------------   215
RAB5C           -----------------------------   216
RAB22A          -----------------------------   194
RAB22B(31)      -----------------------------   194
RAB20           -----------------------------   234
RAB28           -----------------------------   221
RAB29           -----------------------------   203
RAB32           -----------------------------   225
RAB38           -----------------------------   211
RAB34           -----------------------------   259
RAB36           -----------------------------   333
RAB42           -----------------------------   218
RAB39A          -----------------------------   217
RAB39B          -----------------------------   213
RAB7B           -----------------------------   199
RAB7A           -----------------------------   207
RAB9A           -----------------------------   201
RAB9B           -----------------------------   201
RAB23           -----------------------------   237
RAB40C          KGNSLKRSKSIRPPQSPPQNCSRSNCKIS   281
RAB40B          KRSSLRKVKLVRPPQSPPKNCTRNSCKIS   278
RAB40A          K-SSLCKVEIVCPPQSPPKNCTRNSCKIS   277
RAB40AL         KRSSLCKVKIVCPPQSPPKNCTRNSCKIS   278
RAB33A          -----------------------------   237
RAB33B          -----------------------------   229
RAB30           -----------------------------   203
RAB19           -----------------------------   217
RAB43           -----------------------------   212
RAB18           -----------------------------   206
RAB2A           -----------------------------   212
RAB2B           -----------------------------   216
RAB14           -----------------------------   215
RAB4A           -----------------------------   218
RAB4B           -----------------------------   213
RAB25           -----------------------------   213
RAB11A          -----------------------------   216
RAB11B          -----------------------------   218
RAB12           -----------------------------   244
RAB26           -----------------------------   256
RAB37           -----------------------------   223
RAB3D           -----------------------------   219
RAB3B           -----------------------------   219
RAB3A           -----------------------------   220
RAB3C           -----------------------------   227
RAB15           -----------------------------   212
RAB13           -----------------------------   203
RAB10           -----------------------------   200
RAB8A           -----------------------------   207
RAB8B           -----------------------------   207
RAB35           -----------------------------   201
RAB1A           -----------------------------   205
RAB1B           -----------------------------   201
```

Figure S4. Full Sequence alignment of Rab proteins in phylogenetic order compared to Rab27A and B (top). Unique cysteines C123 and C188 are highlighted in red.

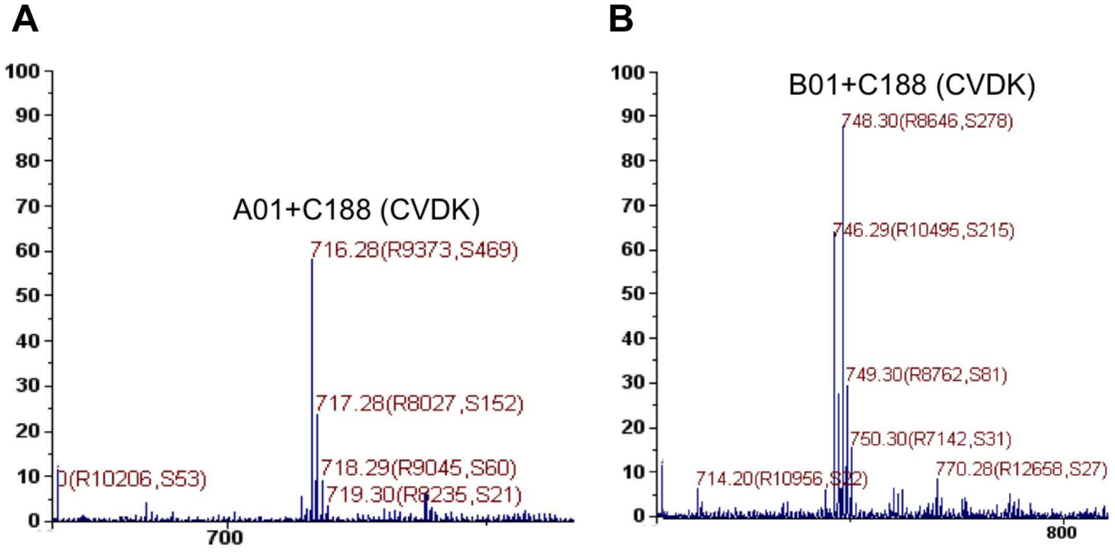

Figure S5. Tryptic digestion and peptide mass fingerprinting for labelling site-ID of A) **A01**-fRab27A-C188 and B) **B01**-fRab27A-C188.

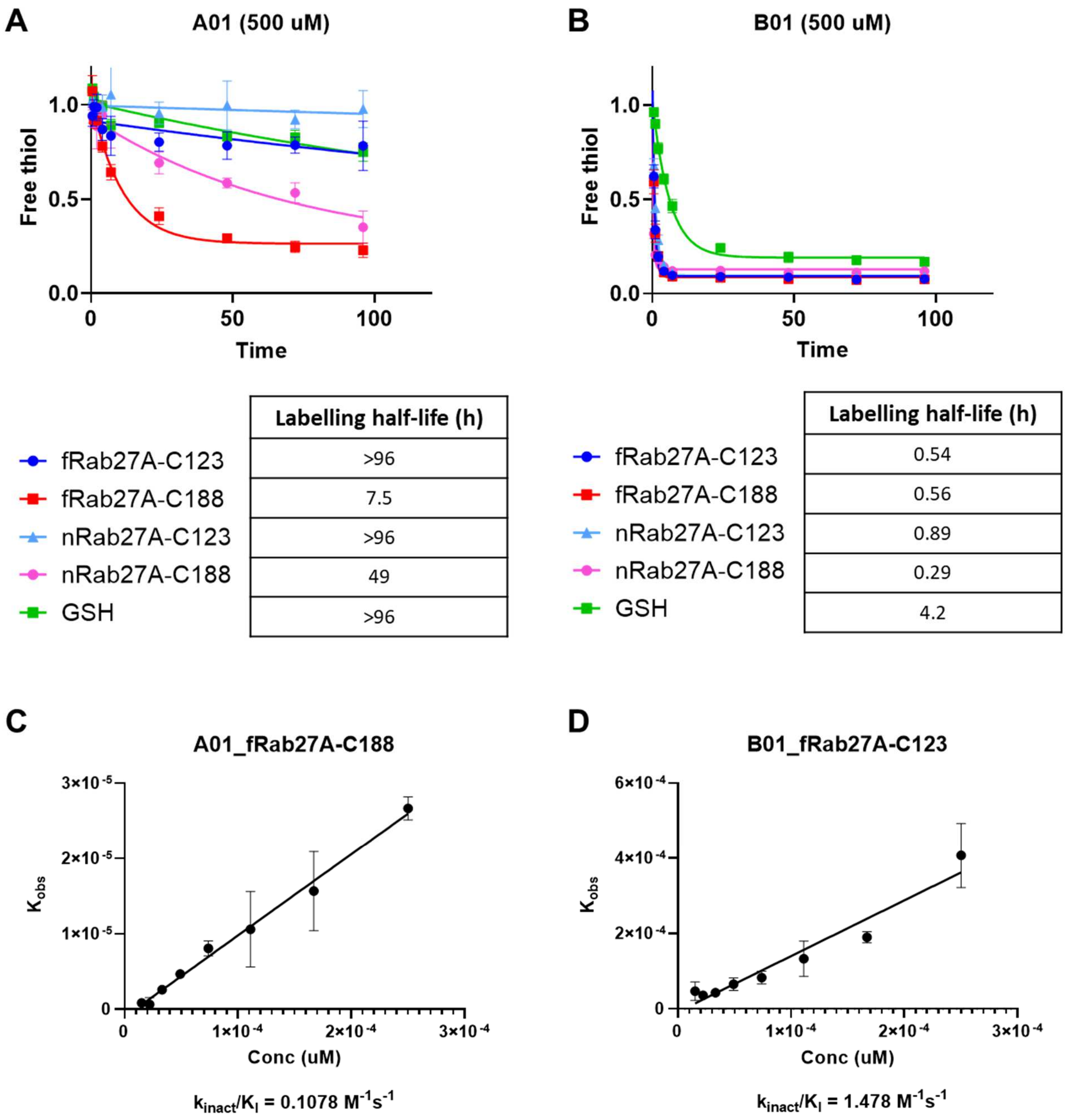

A01 (500 uM)

	Labelling half-life (h)
fRab27A-C123	>96
fRab27A-C188	7.5
nRab27A-C123	>96
nRab27A-C188	49
GSH	>96

B01 (500 uM)

	Labelling half-life (h)
fRab27A-C123	0.54
fRab27A-C188	0.56
nRab27A-C123	0.89
nRab27A-C188	0.29
GSH	4.2

Figure S6. Biochemical characterisation of hits. A–B) qIT data against fRab27A-C123, fRab27A-C188, nRab27A-C123, nRab27A-C188 and GSH including labelling half-lives for resynthesised hits **A01** (A) and **B01** (B). C–D) k_{obs}/[I] graphs for **A01** against fRab27A-C188 (C), and for **B01** against fRab27A-C123 (D)

A

B

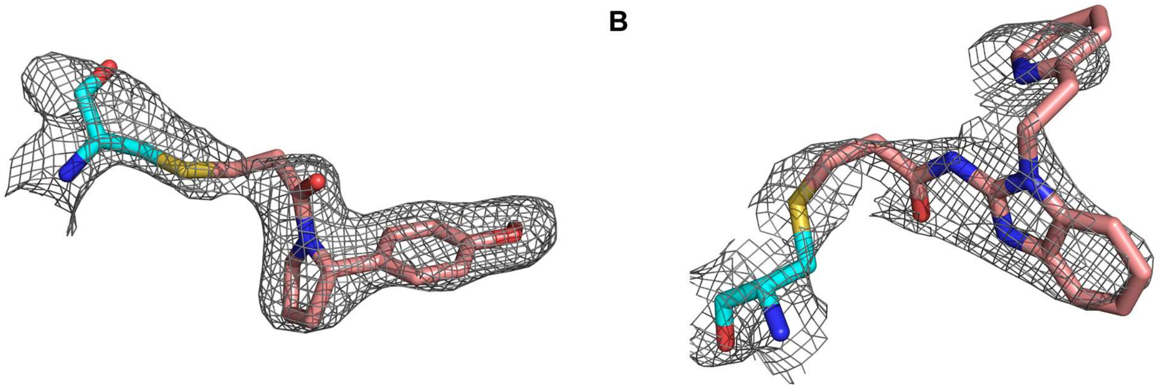

Figure S7. Electron density maps for **A01** and **B01**. $2F_O$-F_C electron density maps (grey) contoured at 1.0 σ for ligands (pink) covalently bound to a cysteine residue (cyan). A) **A01** bound to fRab27A-C188 (full structure shown in **Fig. 3B** and **3C**) and B) **B01** bound to fRab27A-C123 (full structure shown in **Fig. 3E** and **3F**) superimposed on the final model of the respective ligands.

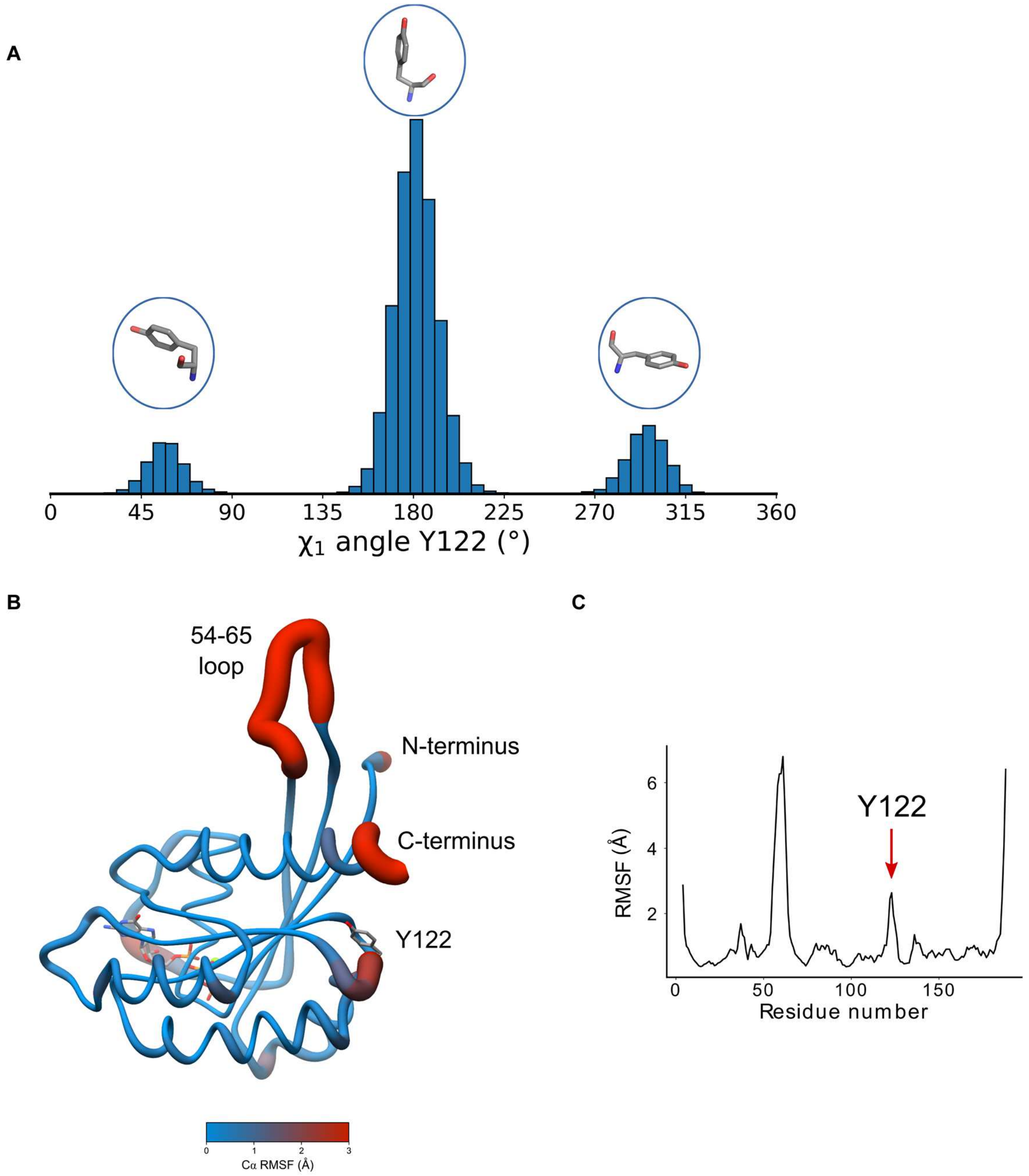

Figure S8. Molecular dynamics and rotameric properties of Y122 in Rab27A. (A) Distribution of chi-1 angles of Y122 during a 250 ns molecular dynamics simulation. (B) Structure of Rab27A, coloured according to the C-alpha Root Mean Squared Fluctuations. Red colours (thicker ribbon) correspond to more mobile regions, whereas blue regions are more rigid. (C) C-alpha Root Mean Squared Fluctuations of Rab27A plotted against its primary sequence.

Supplementary Tables

Table S1. Data Processing and Refinement Statistics for fRab27A

Data Collection	
Space group	$P3_121$
Unit cell parameters (Å)	*a* = 117.71, *b* = 117.71, *c* = 115.67
Wavelength (Å)	0.97949
Resolution (Å)	50.97 - 2.32 (2.40 - 2.32)
Total reflections	80969 (7952)
Unique reflections	40486 (3976)
Multiplicity	2.0 (2.0)
Completeness (%)	99.96 (99.95)
$\langle I \rangle / \langle \sigma(I) \rangle$	20.04 (3.29)
R_{merge}	0.027 (0.240)
R_{meas}	0.039 (0.340)
Wilson *B* factor	36.64
$CC_{1/2}$	0.999 (0.844)
Refinement	
Reflections used in refinement	40483 (3976)
Reflections used for R_{free}	1945 (229)
R_{work} (%)	0.159
R_{free} (%)	0.198
Rmsd bond lengths (Å)	0.008
Rmsd bond angles (°)	1.10
Average *B* factors (Å^2)/Number of atoms	
Macromolecules	42.13/ 3426
Water molecules	47.12/398
Ligand non-H atoms gppnhp-Mg^{2+}	28.3/66
Φ/Ψ angles (%)	
Ramachandran Most favored region (%)	97.79
Ramachandran allowed region (%)	2.1
Ramachandran outliers (%)	0.0
Rotamer outliers (%)	0.56

Statistics for the highest-resolution shell are shown in parentheses.

$R_{merge} = \Sigma(I_{hl} - \langle I_h \rangle)/\Sigma \langle I_h \rangle$

$R_{meas} = \Sigma\sqrt{(n_h/n_h - 1)}(I_{hl} - \langle I_h \rangle)/\Sigma \langle I_h \rangle$

Table S2. Data Processing and Refinement Statistics for fRab27A-C188 covalently bound to **A01**

Data Collection	
Space group	$P2_12_12_1$
Unit cell parameters (Å)	a = 61.71, b = 76.82, c = 117.82
Wavelength (Å)	0.97949
Resolution (Å)	36.52 - 2.23 (2.31 - 2.23)
Total reflections	54026 (3920)
Unique reflections	27128 (2005)
Multiplicity	2.0 (2.0)
Completeness (%)	96.86 (72.64)
$<I>/<\sigma(I)>$	13.67 (2.01)
R_{merge}	0.0340 (0.360)
R_{meas}	0.048 (0.509)
Wilson B factor	41.61
$CC_{1/2}$	1 (0.929)
Refinement	
Reflections used in refinement	27126 (2005)
Reflections used for R_{free}	1342 (98)
R_{work} (%)	0.177
R_{free} (%)	0.224
Rmsd bond lengths (Å)	0.255
Rmsd bond angles (°)	2.82
Average B factors (Å^2)/Number of atoms	
Macromolecules	46.28/3287
Water molecules	50.94/179
Ligand non-H atoms gppnhp-Mg^{2+}	51.70/160
Φ/Ψ angles (%)	
Ramachandran Most favored region (%)	98.02
Ramachandran allowed region (%)	1.98
Ramachandran outliers (%)	0.0
Rotamer outliers (%)	0.00

Statistics for the highest-resolution shell are shown in parentheses.

$R_{merge} = \Sigma(I_{hl} - < I_h >)/\Sigma < I_h >$

$R_{meas} = \Sigma\sqrt{}(n_h/n_h - 1)(I_{hl} - < I_h >)/\Sigma < I_h >$

Table S3. Data Processing and Refinement Statistics for fRab27A-C123 covalently bound to **B01**

Data Collection	
Space group	$P2_12_12_1$
Unit cell parameters (Å)	*a* = 61.38, *b* = 76.66, *c* = 118.24
Wavelength (Å)	0.97949
Resolution (Å)	64.33 - 2.32 (2.40 - 2.32)
Total reflections	49537 (4902)
Unique reflections	24831 (2455)
Multiplicity	2.0 (2.0)
Completeness (%)	99.82 (99.67)
$\langle I\rangle/\langle\sigma(I)\rangle$	9.77 (2.23)
R_{merge}	0.059 (0.325)
R_{meas}	0.083 (0.460)
Wilson *B* factor	32.90
$CC_{1/2}$	0.993 (0.832)
Refinement	
Reflections used in refinement	24809 (2448)
Reflections used for R_{free}	1209 (116)
R_{work} (%)	0.183
R_{free} (%)	0.254
Rmsd bond lengths (Å)	0.008
Rmsd bond angles (°)	1.02
Average *B* factors (Å^2)/Number of atoms	
Macromolecules	35.73/3449
Water molecules	38.33/249
Ligand non-H atoms gppnhp-Mg^{2+}	41.01/176
Φ/Ψ angles (%)	
Ramachandran Most favored region (%)	96.59
Ramachandran allowed region (%)	3.41
Ramachandran outliers (%)	0.0
Rotamer outliers (%)	0.84

Statistics for the highest-resolution shell are shown in parentheses.

$R_{merge} = \Sigma(I_{hl} - \langle I_h \rangle)/\Sigma \langle I_h \rangle$

$R_{meas} = \Sigma\sqrt{(n_h/n_h - 1)}(I_{hl} - \langle I_h \rangle)/\Sigma \langle I_h \rangle$

Table S4. Data from intact mass spectrometry and qIT screen used for hit validation against fRab27A-C123 (top) and fRab27A-C188 (bottom).

fRab27A-C123

	REF	qIT half-life	Mono-modification by intact protein MS	MS half-life	Validated
**CA32/228**	4.2	21.4 h	Yes: expect 250 Da, observed 252	34.3 h	No
**CA84**	2.3004E-12	-	Wrong mass: expect 255 Da, observed 326	-	No
**CA144 (B01)**	2.9	4.7 h	Yes: expect 292 Da, observed 293	6.6 h	**Yes**
**CA92**	4.8	-	Wrong mass: expect 268 Da, observed 352	-	No

fRab27A-C188

	REF	qIT half-life	Mono-modification by intact protein MS	MS half-life	Validated
**CA32/228**	7.6	-	Wrong mass: expect 250 Da, observed 359	-	**No**
**CA84**	2.3	-	Protein degraded	-	**No**
**CA89**	1.6	4.3 h	Yes: expect 284 Da, observed 282	7.4 h	**Yes**
**CA144 (B01)**	2.4	4.5 h	Yes: expect 292 Da, observed 291	11.8 h	**No**
**EL1062 (A01)**	2.5	26.2 h	Yes: expect 231 Da, observed 231	32.5 h	**Yes**
**EL1064**	2.2	12.8 h	Yes: expect 252 Da, observed 253	15.8 h	**Yes**
**CA193**	2	-	Wrong mass: expect 209 Da, observed 261	-	**No**
**CA187**	1.7	4.3 h	Yes: expect 252 Da, observed 250	13.2 h	**No**
**CA53**	5.7	-	No labelling	-	**No**

Materials and Methods

Protein expression and purification

All Rab27A constructs contain the sequence for human Rab27A (UniProt entry P51159, residues 1–192, mutations: Q78L and C123S or C188S or both as specified), which was cloned into a pET15b vector (Invitrogen) including a N-terminal His-tag followed by a Tobacco Etch Virus (TEV) recognition site (ENLYFQ¦G). Fusion constructs also contain the C-terminus of Slp2a SHD1 (SFLTEEEQEAIMKVLQRDAALKRAEEER (residues 5–32)) linked to the N-terminus of Rab27A via a flexible poly glycine-serine linker (GSGSGSG). For protein expression, plasmids were transformed to E. coli BL21 cells and spread on LB agar plates containing 100 mg/L Ampicillin for selection. Single colonies were picked for amplification and incubated overnight into LB media containing 100 mg/L Ampicillin at 37°C, shaking. Big scale cultures were inoculated using these overnight cultures at 1% v/v, and grown at 37 °C until absorbance at 600 nm reached 0.7. Protein expression was induced using 0.5 mM isopropyl β-D-1-thiogalactopyranoside (IPTG) at 37 °C for 3 hours. Subsequently cells were pelleted at 4k rpm for 10 min, then re-suspended in lysis buffer containing 500 mM NaCl, 10 mM imidazole, 5 mM $MgCl_2$ and 50 mM Tris at pH 8.0 Cells were lysed with a cell disruptor at 25K psi and centrifuged at 15k rpm for 45 min. The supernatant was loaded on Ni^{2+}-NTA resin equilibrated with lysis buffer, washed extensively and eluted using buffer containing 500 mM NaCl, 300 mM imidazole, 5 mM $MgCl_2$ and 50 mM Tris at pH 8.0 The protein was dialyzed for 6 h using 100 mM NaCl, 5 mM $MgCl_2$ and 50 mM Tris, pH 8.0 Afterwards TEV protease (obtained in-house as previously described[5]) was added to the protein solution at a molar ratio of 1/20 in the presence of 1 mM DTT, and the solution was incubated overnight at 4 °C, shaking. The solution was dialyzed using 100 mM NaCl, 5 mM $MgCl_2$ and 50 mM Tris, pH 8 and then loaded on Ni_{2+}-NTA resin. The flowthrough was collected, concentrated to 5.5 mg/mL in 150 mM NaCl, 5 mM $MgCl_2$, 20 mM Tris pH 8 buffer. Then a 10x buffer containing 10 mM $ZnCl_2$ and 2 M $(NH_4)_2SO_4$, 4 molar excess of GppNHp and 25 units of Antarctic phosphatase (New England Biolabs) were added to the solution and incubated overnight at 4 °C. Finally the sample was loaded on a superdex S-75 gel filtration column at a flow rate of 1 mL/min. The column was pre-equilibrated with 150 mM NaCl, 5 mM $MgCl_2$, and 20 mM Tris at pH 8 for crystallography. The peaks corresponding to Rab27A constructs were analysed by SDS-PAGE, pooled, concentrated and flash frozen using liquid nitrogen. All Rab27A constructs containing exposed cysteines at C123 or C188 were purified in buffer containing an additional 0.1% β-mercaptoethanol (βME) during Ni^{2+}-NTA steps.

Protein labelling and purification

To a 15 mL falcon tube were added 600 μL of desired construct (100 μM stock), 100 μL of 50% w/v TCEP-agarose beads (ThermoFisher), and 1.8 mL of 100 mM NaCl, 5 mM $MgCl_2$, and 20 mM HEPES pH 8.0. 50 μL of ligand (50 mM stock) were diluted with 2.45 mL of 100 mM NaCl, 5 mM $MgCl_2$, and 20 mM HEPES pH 8.0, followed by centrifugation (2400 rpm, 5 min). The supernatant was added to the protein mixture and incubated at 4 °C. The reaction was monitored as described in the QIT protocol (*v. infra*). When the labelling reached 90%, the labelled protein solution was concentrated to 0.5 mL by using a Vivaspin 20 filter (5000 MWCO). The protein was diluted with 4.5 mL of 100 mM NaCl, 5 mM $MgCl_2$, and 20 mM HEPES pH 8.0 and concentrated again to 0.5 mL (5x), to remove excess compound, then purified by superdex S-75 gel filtration at a flow rate of 1 mL/min in 150 mM NaCl, 5 mM $MgCl_2$, and 20 mM Tris at pH 8 for crystallography.

Protein crystallisation

Pure samples of fRab27A-C188 labelled with **A01** and fRab27A-C123 labelled with **B01** were concentrated to 15 mg/mL, in buffer containing 150 mM NaCl, 5 mM $MgCl_2$, and 20 mM Tris at pH 8.0. Crystals were grown at 4 °C using the sitting-drop vapor-diffusion method with a mother liquor containing 120 mM $MgCl_2$, 50 mM bis-Tris, and 15% 2-propanol, pH 6.8.

Crystal diffraction, data collection and data processing

Data collections were carried out at i02 beamline in Diamond Light Source (Oxford, UK) at 100*K* of temperature, wavelength 0.9795 Å, and using a Pilatus detector. Data were collected at 0.2°-0.5° oscillations per image and 200° total oscillation per crystal. Data was integrated, scaled and reduced using DIALS.[6] Initial phases were calculated using the molecular replacement program Phaser.[7] The coordinates of Rab27A from chain A of the Rab27A-Slp2a complex (PDB:3BC1) without the nucleotide and the magnesium ion were used as the search model. Subsequently, the initial model generated by phaser was refined through an iterative cycle using COOT[9] and REFMAC5.[10] Final model structures were validated using the Molprobity server[11] at http://molprobity.biochem.duke.edu. All structure images were prepared using Pymol (DeLano Scientific LLC, http://pymol.sourceforge.net/). X-ray data collection, processing and refinement statistics are given in Table S1.

Molecular dynamics simulations

The Rab27A structure (PDB: 3BC1) was simulated with bound GTP and Mg^{2+}. The structure was parametrised using the latest CHARMM36 force field[4], solvated with Tip3p waters[12] and neutralised with Na^+ and Cl^- ions at a concentration of 150 mM. Temperature was coupled for 100 ps at 300 K with the V-rescale method,[13] with positional restraints on the protein heavy atoms. Pressure was then coupled at 1 bar for another 100 ps with position restraints, using the Berendsen algorithm.[14] The Particle mesh Ewald method[15] was used for electrostatic interactions, and LINCS[16] to define the constraints. The integration timestep was 2 fs. The final production simulation was extended for 250 ns. The simulation and data analysis were carried out using the GROMACS simulation package.[17]

qIT assay for screening and hit validation

126 electrophilic acrylamides (see supplementary excel file) were screened in the qIT assay adapted from Craven *et al*[18]. Briefly, the reaction buffer (20 mM HEPES pH 8.0, 100 mM NaCl, 5 mM $MgCl_2$) and quench buffer (20 mM HEPES pH 7.4, 100 mM NaCl, 5 mM $MgCl_2$) were prepared, filtered, de-gassed, and re-gassed with Ar for 15 min on ice. Reaction setup: To each well of a 96-well PCR plate (reaction plate), 8 µL of 50% w/v TCEP-agarose beads in reaction buffer was added, followed by the addition of 92 µL of 10.87 µM protein or glutathione (GSH). In a separate 96-well PCR plate (ligand plate), 3 µL of DMSO or 50 mM ligand in DMSO was added to 147 µL reaction buffer and centrifuged (1k rpm, 5 min, 4 °C). 100 µL of ligand solution or DMSO control from the ligand plate was added to the reaction plate (final concentration: 5 µM protein/GSH and 500 µM ligand). After mixing, the TCEP-agarose beads were pelleted by centrifugation (1k rpm, 5 min, 4 °C) and the plate was kept at 4 °C.

At a series of time points (t = 0.25. 1, 2, 4, 7, 24, 48, 72, and 96 h), a 3 µL aliquot in duplicate from the reaction plate was quenched in a black 384-well plate, in which each well was pre-filled with 27 µL of 7-Diethylamino-3-(4'-Maleimidylphenyl)-4-Methylcoumarin (CPM) solution (1.4 µM in quench buffer). The fluorescence plate was spun down (1k rpm, 1 min) and incubated for 60 min at room temperature and then fluorescence intensity (excitation/emission: 384/470 nm) was measured on an EnVision™ plate reader.
Data analysis: All analyses were conducted using Prism 9.0 software (Graphpad). Each fluorescence readout was normalized to the average of the DMSO controls. The normalized fluorescence was plotted against time. A one phase exponential decay was fitted to each plot (constraints: Y(0) > 0.8; 0 < plateau < 0.3; k > 0). Data from at least three independent assay replicates were used to generate the graphs in Fig. S5.

qIT assay for k_{inact}/K_I determination

The k_{inact}/K_I values were determined from data obtained performing the qIT assay at different compound concentrations (eight 1:1.5 dilutions starting from 250 µM) at room temperature, quenching at different time-points (t= 10, 20, 30, 60, 120, 180, 240, 360, 1440 min). Kinetic curves of thiol labelling over time were used to estimate k_{obs} values, which were then plotted against inhibitor concentration. The resulting linear data were analysed by linear regression to obtain k_{inact}/K_I values.

Peptide mass fingerprint analysis

5 µg labelled or unlabelled recombinant Rab27A construct were run on a 12% SDS-PAGE gel and stained by Coomassie Blue. The expected band was excised and washed in 150 µL of 50% v/v MeCN/H_2O for 5 min at rt, shaking. The supernatant was discarded, and the solid was washed with 150 µL of 50% v/v MeCN/50 mM NH_4HCO_3 for 30 min at rt, shaking. The supernatant was discarded, and the solid was washed with 150 µL of 50% v/v MeCN/10 mM NH_4HCO_3 for 30 min at rt, shaking. The supernatant was dried *in vacuo* for 30 min at 45 °C, then 15 µL of Trypsin (20 µg/100 µL in 50 mM NH_4HCO_3) were added. After 10 min at rt, the mixture was diluted with 15 µL of 10 mM NH_4HCO_3 and incubated overnight at 37 °C, shaking. The supernatant was diluted 1:1 with α-Cyano-4-hydroxycinnamic acid (10 mg/mL in 50% v/v MeCN/H_2O with 0.1% TFA) and anlysed by MALDI-QTOF.

Chemical Synthesis

Abbreviations

DMF (dimethylformamide), EtOAc (ethyl acetate), FCC (flash column chromatography), rt (room temperature), TFA (trifluoroacetic acid), THF (tetrahydrofuran), TLC (thin layer chromatography)

General Information

All chemicals were purchased from Sigma-Aldrich, Apollo Scientific, Acros Organics, Alfa Aesar and used without further purification unless otherwise indicated. All reactions were monitored by thin layer chromatography (TLC) using UV for visualisation unless otherwise stated. Compounds were purified using either an automated system using pre-packed silica cartridges with UV detection or by manual columns using an appropriate solvent mixture as detailed. ^{1}H and ^{13}C NMR spectra were recorded on 400 MHz and 101 MHz respectively Bruker AV instruments at room temperature unless specified otherwise and were referenced to residual solvent signals. Data are presented as follows: chemical shift in ppm, multiplicity (br = broad, app = apparent, s = singlet, d = doublet, t = triplet, q = quartet, p = pentet, m = multiplet), coupling constants in Hz, integration, and rotameric conformation if applicable. High-resolution mass spectrometry (HRMS) and intact mass spectrometry data were obtained by the Imperial Mass Spectrometry facility. m/z values are reported in Daltons (Da) to the nearest 0.0001 Da.

Scheme S1. *Synthesis of hit fragment **A01***

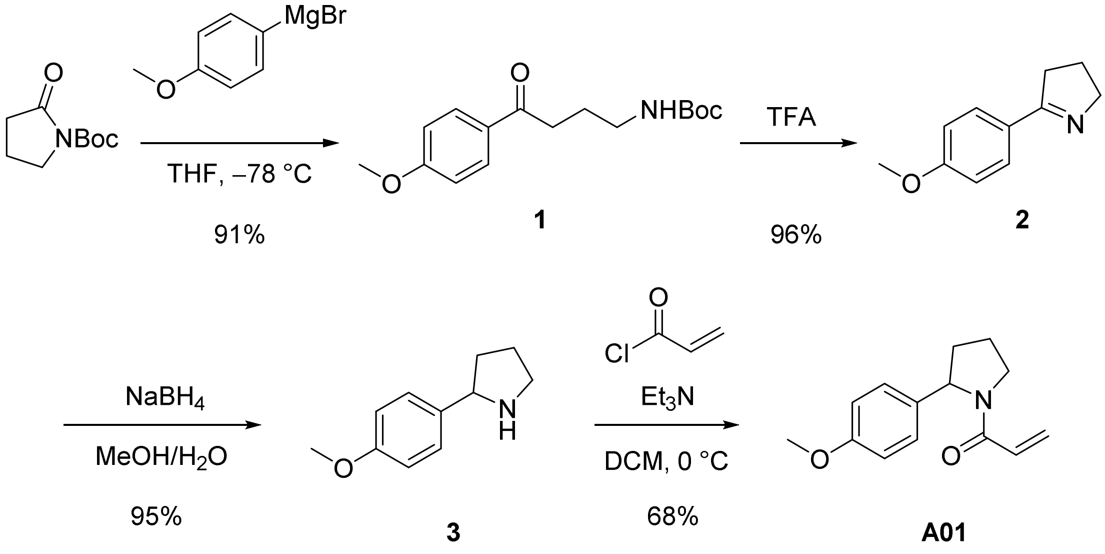

tert-butyl (4-(4-methoxyphenyl)-4-oxobutyl)carbamate (1)

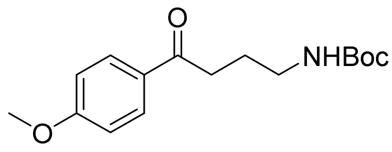

(4-Methoxyphenyl)magnesium bromide (0.5 M in THF, 24 mL, 12 mmol) was added dropwise over 30 min at –78 °C to a stirred solution of *tert*-butyl 2-oxopyrrolidine-1-carboxylate (1.7 mL, 10 mmol) in THF (40 mL). The reaction was stirred for 1 h at –78 °C, then slowly warmed to rt and stirred for 1 h before the pH was adjusted to 1–3 using 1 M HCl. The solution was concentrated *in vacuo*, then diluted with CH_2Cl_2 (70 mL) and $NaHCO_3$ (70 mL), and the aqueous layer extracted with CH_2Cl_2 (3× 50 mL). The combined organic layers were dried over $MgSO_4$, filtered, concentrated *in vacuo*. The crude product was purified by FCC (6%–60% EtOAc/hexane) to give the title compound **1** as a white amorphous solid (2.7 g, 91%).

^{1}H NMR (400 MHz, $CDCl_3$) δ 7.95–7.92 (2H, m), 6.94-6.91 (2H, m), 4.67 (br, 1H, NH), 3.87 (3H, s), 3.24–3.19 (2H, m), 2.97 (2H, t, *J* = 7.2 Hz), 1.92 (2H, p, *J* = 7.0 Hz), 1.42 (9H, s) ppm

^{13}C NMR (101 MHz, $CDCl_3$) δ 198.5, 163.6, 156.2, 130.4, 130.1, 113.9, 79.3, 55.6, 40.4, 35.5, 28.5, 24.8 ppm

5-(4-methoxyphenyl)-3,4-dihydro-2*H*-pyrrole (2)

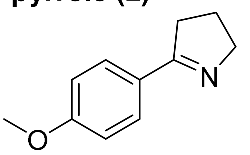

Ketone **1** (0.59 g, 2.0 mmol) was stirred in neat TFA (1.5 mL) at rt for 3.5 h. After the reaction was complete by TLC, the mixture was cooled to 0 °C and 50% w/v NaOH solution was added to the mixture until pH 13–14. The aqueous layer was extracted with CH_2Cl_2 (3x 25 mL), then the combined organic layers were dried over $MgSO_4$, filtered, and concentrated *in vacuo* to afford the title compound **2** as a white crystalline solid which was used without further purification (0.34 g, 96%).

^{1}H NMR (400 MHz, $CDCl_3$) δ 7.82–7.78 (2H, m), 6.94–6.90 (2H, m), 4.04 (2H, tt, *J* = 7.3, 1.9 Hz), 3.84 (3H, s), 2.95–2.90 (2H, m), 2.06–1.99 (2H, m) ppm

^{13}C NMR (101 MHz, $CDCl_3$) δ 172.9, 161.5, 129.4, 127.5, 113.9, 61.4, 55.5, 35.0, 22.8 ppm

HRMS (ES) *m/z* Calculated for $C_{11}H_{14}NO$ $[M+H]^+$ 176.1075, found 176.1080 (Δ 2.8 ppm)

2-(4-methoxyphenyl)pyrrolidine (3)

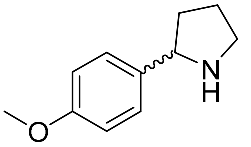

To a stirring solution of imide **2** (0.30 g, 1.7 mmol) in MeOH/H_2O 4:1 (2.0 mL) was added $NaBH_4$ (78 mg, 2.0 mmol) and the reaction was stirred for 20 h at rt. Additional $NaBH_4$ (20 mg, 0.8 mmol) was added and the reaction was stirred until completion as monitored by TLC. The reaction mixture was acidified with 1 M HCl to pH 1–3 and stirred for an additional 30 min, then 1 M NaOH was added until pH 13–15. The aqueous layer was extracted with CH_2Cl_2 (3x 10 mL). The combined organic layers were dried over $MgSO_4$, filtered, and concentrated *in vacuo* to give the title compound **3** as a yellow oil (81 mg, 95%), which was used without further purification.

^{1}H NMR (400 MHz, $CDCl_3$) δ 7.30–7.26 (2H, m), 6.87–6.84 (2H, m), 4.05 (1H, t, *J* = 7.8 Hz), 3.79 (3H, s), 3.19 (1H, ddd, *J* = 10.3, 7.8, 5.3 Hz), 2.98 (1H, ddd, *J* = 10.3, 8.4, 6.6 Hz), 2.24 (1H, br s), 2.18–2.11 (1H, m), 1.99–1.78 (2H, m), 1.72–1.59 (1H, m) ppm

^{13}C NMR (100 MHz, $CDCl_3$) δ 158.6, 136.6, 127.8, 113.9, 62.3, 55.4, 47.0, 34.3, 25.7 ppm

HRMS (ES) *m/z* Calculated for $C_{11}H_{16}NO$ $[M+H]^+$ 178.1232, found 178.1232 (Δ 0.0 ppm)

1-(2-(4-methoxyphenyl)pyrrolidin-1-yl)prop-2-en-1-one (A01)

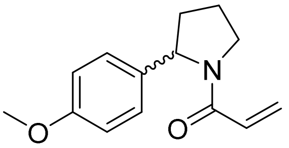

To a stirred solution of amine **3** (89 mg, 0.50 mmol) and Et_3N (0.10 mL, 0.75 mmol) in CH_2Cl_2 (1.5 mL) at 0 °C was added acryloyl chloride (49 µL, 0.60 mmol) in CH_2Cl_2 (0.50 mL) dropwise. The reaction was allowed to warm to rt, stirred for 2 h, diluted with CH_2Cl_2 (10 mL) and then quenched by slow addition of $NaHCO_3$ (10 mL). The aqueous layer was extracted with CH_2Cl_2 (2× 10 mL) then the combined organic layers were dried over $MgSO_4$, filtered, and concentrated *in vacuo*. Purification by FCC (12%–100% EtOAc/hexanes) afforded the title compound **A01** as a clear, colourless oil (79 mg, 68%).

^{1}H NMR (400 MHz, $CDCl_3$) *Rotameric ratio maj:min 0.76:0.24*; δ 7.09–7.03 (2H, m), 6.86–6.79 (2H, m), 6.53 (1H, dd, *J* = 16.8, 10.3 Hz, min. rot.), 6.34 (1H, dd, *J* = 16.8, 2.1 Hz, maj. rot.), 6.29 (1H, dd, *J* = 16.8, 2.2 Hz, maj. rot.), 6.12 (1H, dd, *J* = 16.7, 10.3 Hz, maj. rot.), 5.66 (1H, dd, *J* = 10.3, 2.2 Hz, min. rot.), 5.44 (1H, dd, *J* = 10.2, 2.2 Hz, maj. rot.), 5.21 (1H, dd, *J* = 8.0, 3.2 Hz, min. rot.), 5.00 (1H, dd, *J* = 7.8, 2.0 Hz, maj. rot.), 3.83–3.78 (1H, m), 3.77 (3H, s, maj. rot.), 3.74 (3H, s, min. rot.), 3.72–3.65 (1H, m), 2.38–2.18 (1H, m), 2.01–1.80 (3H, m) ppm

^{13}C NMR (100 MHz, $CDCl_3$) δ 165.4 (maj. rot.), 164.4 (min. rot.), 158.8 (maj. rot.), 158.4 (min. rot.), 135.4 (maj. rot.), 135.0 (min. rot.), 129.0 (maj. rot.), 128.8 (min. rot.), 128.0 (min. rot.), 127.4 (maj. rot.), 126.7 (min. rot.), 126.6 (maj. rot.), 114.1 (maj. rot.), 113.9 (min. rot.), 60.8 (maj. rot.), 60.2 (min. rot.), 55.3, 47.6 (min. rot.), 47.1 (maj. rot.), 36.4 (maj. rot.), 34.0 (min. rot.), 23.9 (min. rot.), 21.6 (maj. rot.) ppm

HRMS (ES) *m/z* Calculated for $C_{14}H_{18}NO_2$ $[M+H]^+$ 232.1338, found 232.1340 (Δ 0.9 ppm)

Scheme S2. *Synthesis of hit fragment **B01***

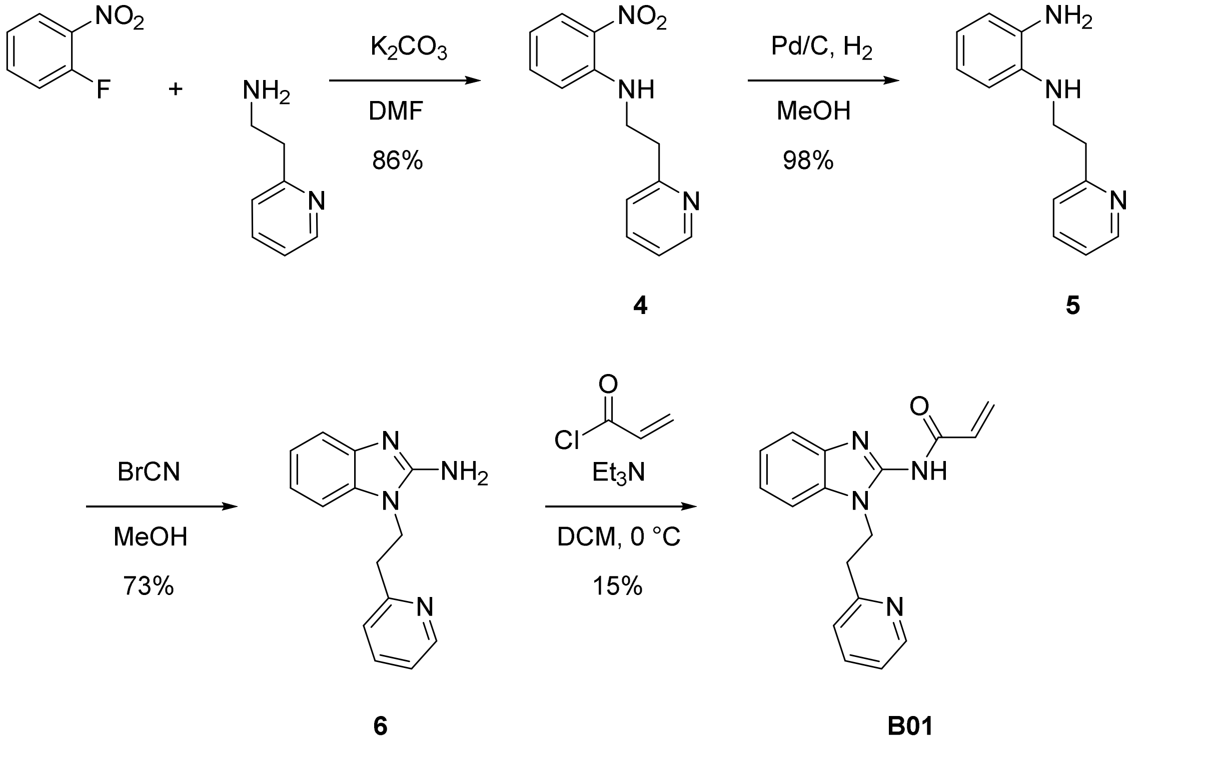

2-nitro-*N*-(2-(pyridin-2-yl)ethyl)aniline (4)

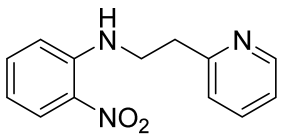

1-Fluoro-2-nitrobenzene (0.42 mL, 4.0 mmol), 2-(2-aminoethyl)pyridine (0.48 mL, 4.0 mmol) and K_2CO_3 (1.1 g, 8.0 mmol) were dissolved in DMF (10 mL) and stirred at rt for 24 h. The reaction mixture was then diluted with EtOAc (50 mL) and $NaHCO_3$ (50 mL). The aqueous layer was extracted with EtOAc (3× 50 mL), then the combined organic layers were washed with 5% LiCl (30 mL) and brine (30 mL), dried over $MgSO_4$, filtered and concentrated *in vacuo*. Purification by

FCC (12%–100% EtOAc/hexane) afforded the title compound **4** as a bright orange oil (0.83 g, 86%).

^{1}H NMR (400 MHz, $CDCl_3$) δ 8.58 (1H, ddd, *J* = 4.9, 1.8, 0.9 Hz), 8.29 (1H, br s, NH), 8.12 (1H, dd, *J* = 8.7, 1.7 Hz), 7.61 (1H, dt, *J* = 7.7, 1.8 Hz), 7.40 (1H, ddd, *J* = 8.6, 7.0, 1.6 Hz), 7.20 (1H, d, *J* = 7.7 Hz), 7.15 (1H, ddd, *J* = 7.5, 4.9, 1.1 Hz), 6.90 (1H, d, *J* = 8.5 Hz), 6.60 (1H, ddd, *J* = 8.5, 6.9, 1.2 Hz), 3.73 (2H, dt, *J* = 6.8, 5.3 Hz), 3.17 (2H, t, *J* = 6.8 Hz) ppm

^{13}C NMR (101 MHz, $CDCl_3$) δ 158.5, 149.8, 145.4, 136.8, 136.3, 132.0, 126.9, 123.5, 121.9, 115.3, 113.8, 42.7, 37.3 ppm

HRMS (ES) *m/z* Calculated for $C_{13}H_{14}N_3O_2$ $[M+H]^+$ 244.1086, found 244.1090 (Δ 1.6 ppm)

***N*^{1}-(2-(pyridin-2-yl)ethyl)benzene-1,2-diamine (5)**

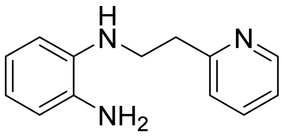

Pyridine **4** (0.65 mg, 2.7 mmol) was dissolved in MeOH (6.0 mL) and Pd/C (10%, 65.0 mg) was added. The reaction mixture was degassed and flushed with H_2 three times, then stirred at rt until reaction was complete by TLC. The reaction was filtered through celite to afford the title compound **5** as a dark brown oil (0.56 g, 98%), which was used without further purification.

^{1}H NMR (400 MHz, $CDCl_3$) δ 8.57 (1H, d, *J* = 4.6 Hz), 7.60 (1H, t, *J* = 7.7 Hz), 7.18 (1H, d, *J* = 7.8 Hz), 7.14 (1H, t, *J* = 6.2 Hz), 6.85–6.81 (1H, m), 6.73 (1H, d, *J* = 7.9 Hz), 6.71–6.66 (2H, m), 3.64 (3H, br), 3.53 (2H, t, *J* = 6.7 Hz), 3.14 (2H, t, *J* = 6.7 Hz) ppm

^{13}C NMR (101 MHz, $CDCl_3$) δ 159.9, 149.3, 137.4, 136.5, 134.6, 123.3, 121.5, 120.4, 118.7, 116.2, 112.0, 44.0, 37.5 ppm

HRMS (ES) *m/z* Calculated for $C_{13}H_{16}N_3$ $[M+H]^+$ 214.1344, found 214.1346 (Δ 0.2 ppm)

1-(2-(pyridin-2-yl)ethyl)-1*H*-benzo[d]imidazol-2-amine (6)

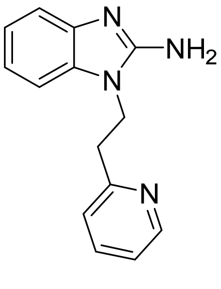

Aniline **5** (0.57 mg, 2.7 mmol) was dissolved in MeOH (20 mL), then cyanogen bromide (0.60 g, 4.0 mmol) was added to the solution. The reaction was stirred at rt for 2 h, and then concentrated *in vacuo*. The residue was diluted with EtOAc (60 mL) and 1 M NaOH (50 mL), and the aqueous layer extracted with EtOAc (3× 60 mL). The combined organic layers were washed with brine (50 mL), dried over Na_2SO_4, filtered and concentrated *in vacuo*. Purification by FCC (5% MeOH/CH_2Cl_2, 0.5% NH_4OH) afforded the title compound **6** as a purple-grey powder (0.46 g, 73%).

R_f 0.39 (10% MeOH/CH_2Cl_2, 0.5% NH_4OH)

^{1}H NMR (400 MHz, $CDCl_3$) δ 8.54 (1H, ddd, *J* = 4.9, 1.6, 0.7 Hz), 7.51 (1H, dt, *J* = 7.7, 1.8 Hz), 7.36 (1H, m), 7.16–7.03 (4H, m), 6.98 (1H, d, *J* = 7.9 Hz), 5.41 (2H, br), 4.48 (2H, t, *J* = 6.0 Hz), 3.32 (2H, t, *J* = 6.1 Hz) ppm

^{13}C NMR (101 MHz, $CDCl_3$) δ 157.4, 154.5, 149.0, 141.8, 137.1, 134.1, 124.4, 122.3, 121.6, 119.7, 116.2, 107.5, 40.7, 36.6 ppm

HRMS (ES) *m/z* Calculated for $C_{14}H_{15}N_4$ $[M+H]^+$ 239.1297, found 239.1297 (Δ 0.0 ppm)

***N*-(1-(2-(pyridin-2-yl)ethyl)-1*H*-benzo[*d*]imidazol-2-yl)acrylamide (B01)**

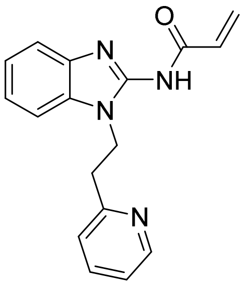

Acroyl chloride (31 µL, 0.39 mmol) was dissolved in CH_2Cl_2 (1.0 mL) and the resulting solution was added dropwise to a stirring solution of benzimidazole **6** (92 mg, 0.39 mmol) and Et_3N (81 µL, 0.58 mmol) in CH_2Cl_2 (1.0 mL) at 0 °C. The reaction was then allowed to warm to rt, stirred for 2 h, diluted with CH_2Cl_2 (10 mL) and then quenched by addition of $NaHCO_3$ (10 mL). The aqueous layer was extracted with CH_2Cl_2 (3× 10 mL), then the combined organic layers were dried over $MgSO_4$, filtered, and concentrated *in vacuo*. Purification by FCC (25%–100% EtOAc/hexane) afforded the title compound as a white solid (9.6 mg, 10%).

^{1}H NMR (400 MHz, $CDCl_3$) δ 12.30 (s, 1H), 8.58 (ddd, *J* = 4.9, 1.9, 0.9 Hz, 1H), 7.51 (td, *J* = 7.7, 1.9 Hz, 1H), 7.31–7.25 (m, 1H), 7.21–7.10 (m, 4H), 7.06 (dt, *J* = 7.8, 1.1 Hz, 1H), 6.47 (d, *J* = 6.0 Hz, 2H), 5.76–5.64 (m, 1H), 4.59 (dd, *J* = 7.6, 6.7 Hz, 2H), 3.33 (t, *J* = 7.1 Hz, 2H) ppm

^{13}C NMR (101 MHz, $CDCl_3$) δ 176.5, 158.0, 153.5, 149.5, 137.1, 136.5, 129.6, 126.0, 123.7, 122.9, 122.8, 121.9, 111.2, 109.4, 42.0, 36.7 ppm

HRMS (ES) *m/z* Calculated for $C_{12}H_{15}N_4$ $[M+H]^+$ 215.1297, found 215.1297 (Δ 0.0 ppm)

NMR spectra for A01 and B01

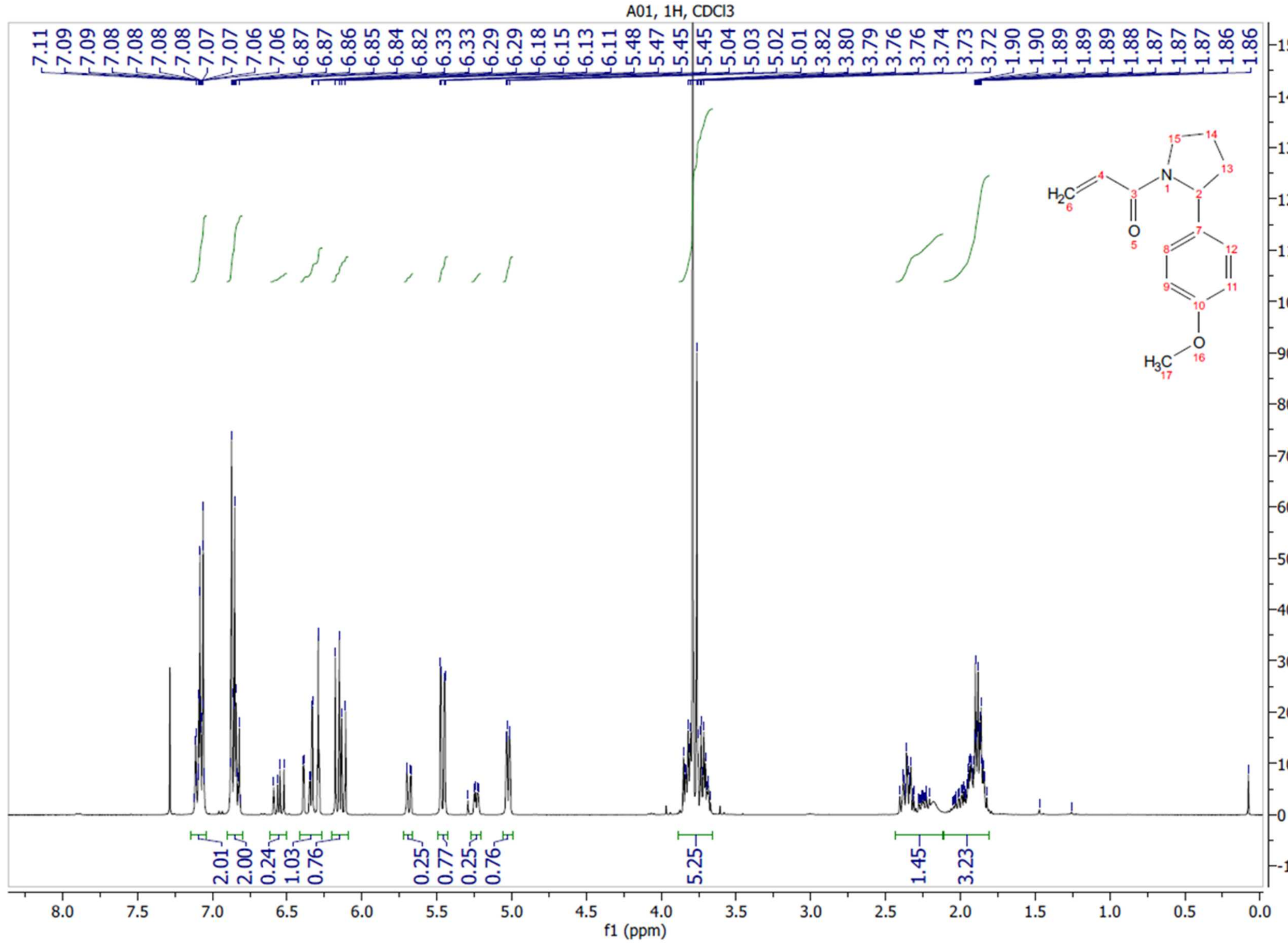
A01, 1H, CDCl3
7.11 7.09 7.09 7.08 7.08 7.08 7.08 7.07 7.07 7.06 7.06 6.87 6.87 6.86 6.85 6.84 6.82 6.33 6.33 6.29 6.29 6.18 6.15 6.13 6.11 5.48 5.47 5.45 5.45 5.04 5.03 5.02 5.01 3.82 3.80 3.79 3.76 3.76 3.74 3.73 3.72 1.90 1.90 1.89 1.89 1.89 1.88 1.87 1.87 1.87 1.86 1.86
2.01 2.00 0.24 1.03 0.76 0.25 0.77 0.25 0.76 5.25 1.45 3.23
f1 (ppm)
H2C O N H3C O

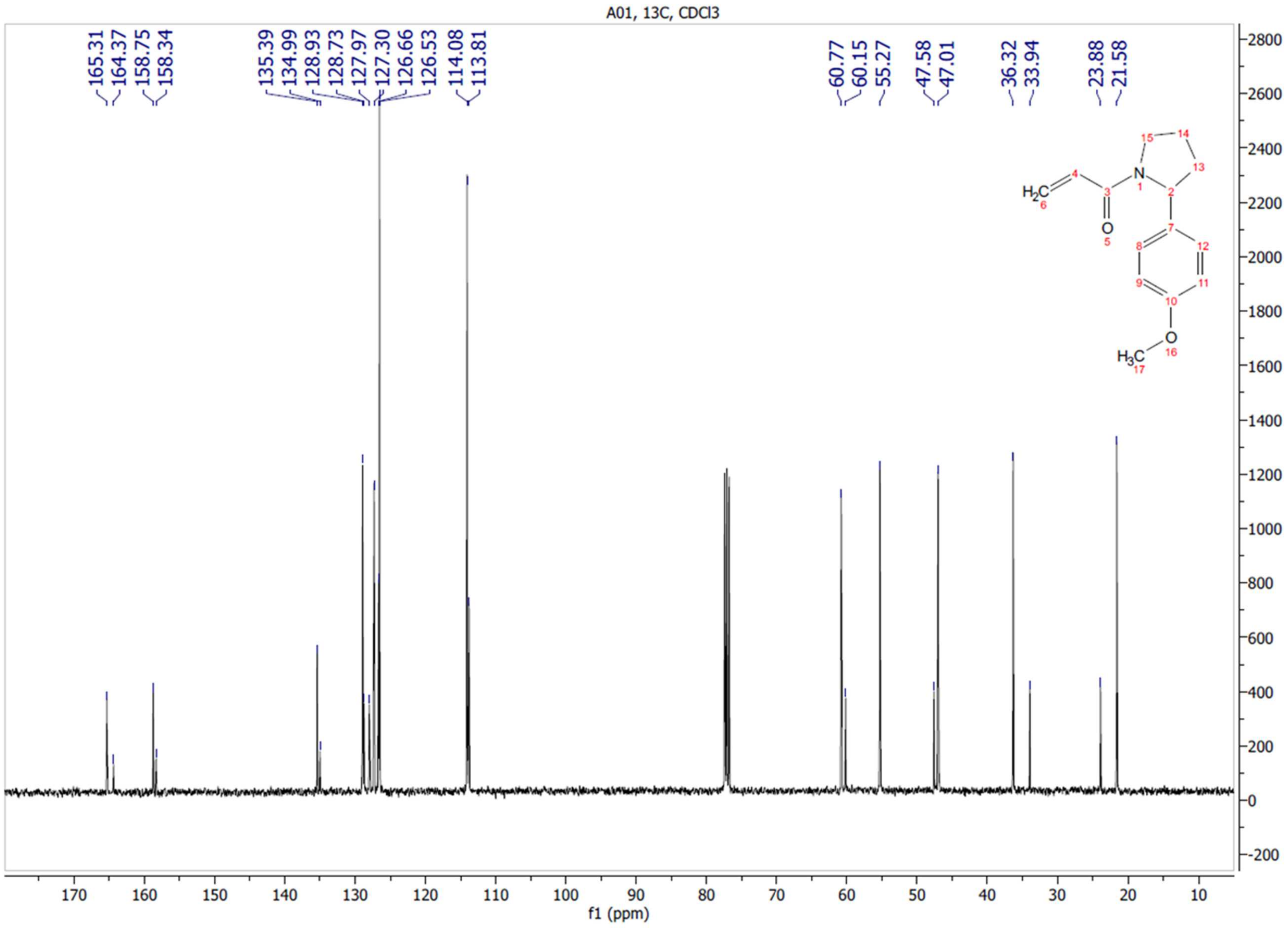
A01, 13C, CDCl3
165.31
164.37
158.75
158.34
135.39
134.99
128.93
128.73
127.97
127.30
126.66
126.53
114.08
113.81
60.77
60.15
55.27
47.58
47.01
36.32
33.94
23.88
21.58
f1 (ppm)
H2C
O
N
H3C
O

B01, 1H, CDCl3

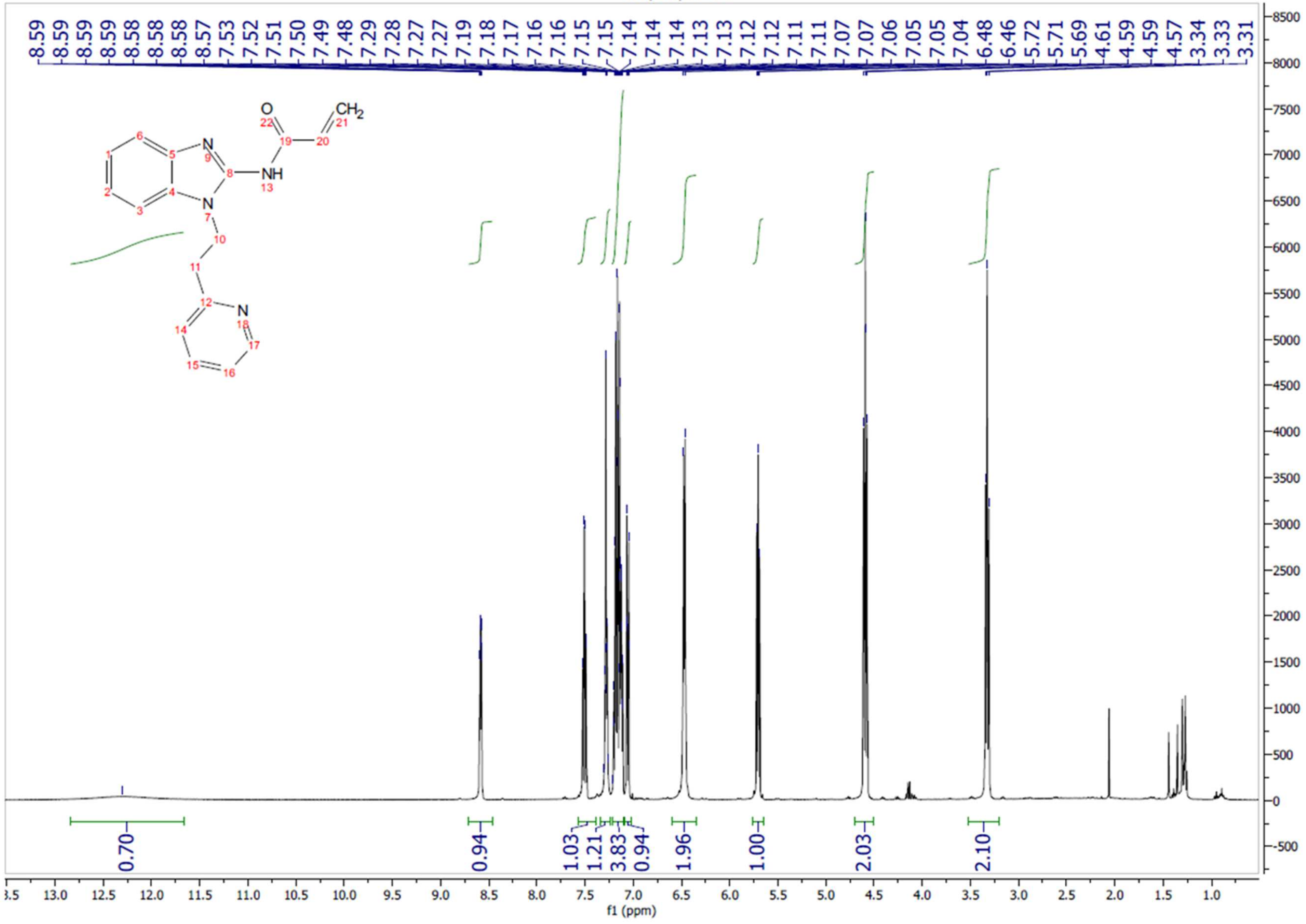
8.59
8.59
8.59
8.59
8.58
8.58
8.58
8.57
7.53
7.52
7.51
7.50
7.49
7.48
7.29
7.28
7.27
7.27
7.19
7.18
7.17
7.16
7.16
7.15
7.15
7.14
7.14
7.14
7.13
7.13
7.12
7.12
7.11
7.11
7.07
7.07
7.06
7.05
7.05
7.04
6.48
6.46
5.72
5.71
5.69
4.61
4.59
4.59
4.57
3.34
3.33
3.31
O
CH2
NH
N
0.70
0.94
1.03
1.21
3.83
0.94
1.96
1.00
2.03
2.10
f1 (ppm)

B01, 13C, CDCl3

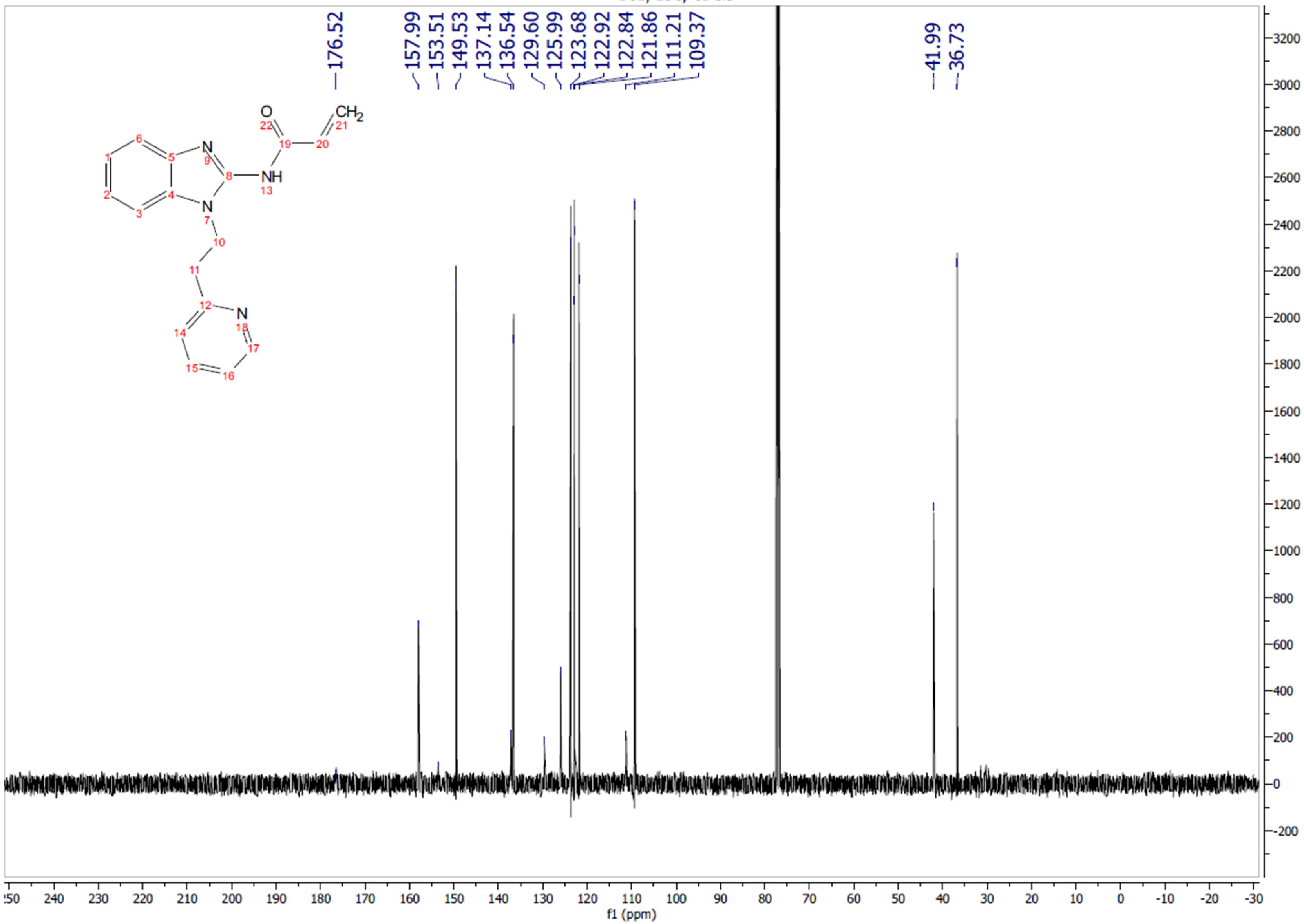

Supplementary References

(1) Kozakov, D.; Grove, L. E.; Hall, D. R.; Bohnuud, T.; Mottarella, S. E.; Luo, L.; Xia, B.; Beglov, D.; Vajda, S. The FTMap Family of Web Servers for Determining and Characterizing Ligand-Binding Hot Spots of Proteins. *Nat. Protoc.* **2015**, 10 (5), 733–755.

(2) Joung, J. Y.; Lee, H. Y.; Park, J.; Lee, J.-Y.; Chang, B. H.; No, K. T.; Nam, K.-Y.; Hwang, J. S. Identification of Novel Rab27a/Melanophilin Blockers by Pharmacophore-Based Virtual Screening. *Appl. Biochem. Biotechnol.* **2014**, 172 (4), 1882–1897.

(3) Johnson, J. L.; Ramadass, M.; He, J.; Brown, S. J.; Zhang, J.; Abgaryan, L.; Biris, N.; Gavathiotis, E.; Rosen, H.; Catz, S. D. Identification of Neutrophil Exocytosis Inhibitors (Nexinhibs), Small Molecule Inhibitors of Neutrophil Exocytosis and Inflammation: Druggability of the Small GTPase Rab27a. *J. Biol. Chem.* **2016**, 291 (50), 25965–25982.

(4) Baell, J.; Walters, M. A. Chemistry: Chemical Con Artists Foil Drug Discovery. *Nature* **2014**, 513 (7519), 481–483.

(5) Tropea, J. E.; Cherry, S.; Waugh, D. S. Expression and Purification of Soluble His6-Tagged TEV Protease. *Methods Mol. Biol.* **2009**, 498, 297–307.

(6) Beilsten-Edmands, J., Winter, G., Gildea, R., Parkhurst, J., Waterman, D., and Evans, G. Scaling diffraction data in the DIALS software package: algorithms and new approaches for multi-crystal scaling. *Acta Crystallogr.* **2020**, Sect. D76, 385–399.

(7) McCoy, A. J., Grosse-Kunstleve, R. W., Adams, P. D., Winn, M. D., Storoni, L. C., and Read, R. J. “Phaser crystallographic software,” *J. Appl. Crystallogr.* **2007**, 40 (4), 658–674.

(8) Emsley, P., Lohkamp, B., Scott, W. G., and Cowtan, K. Features and development of Coot, *Acta Crystallogr.* ***2010,*** Sect. D Biol. Crystallogr., 66 (4), 486–501.

(9) Murshudov, G. N., Skubák, P., Lebedev, A. A., Pannu, N. S., Steiner, R. A., Nicholls, R. A., Winn, M. D., Long, F., Vagin, A. A. REFMAC 5 for the refinement of macromolecular crystal structures, *Acta Crystallogr.*, **2011**, Sect. D Biol. Crystallogr., 67 (4), 355–367.

(10) V. B. Chen et al., MolProbity: All-atom structure validation for macromolecular crystallography, *Acta Crystallogr.* **2010**, Sect. D Biol. Crystallogr., 66 (1), 12–21.

(11) Huang, J., Rauscher, S., Nawrocki, G., Ran, T., Feig, M., de Groot, B. L., Grubmüller, H., MacKerell, A. D. JrCHARMM36m: an improved force field for folded and intrinsically disordered proteins. *Nature Methods* **2017**, 14, 71–73.

(12) Jorgensen, W. L., Chandrasekhar, J., Madura, J. D., Impey, R. W. & Klein, M. L. Comparison of simple potential functions for simulating liquid water. *J Chem Phys* **1983**, 79, 926.

(13) Bussi, G., Donadio, D. & Parrinello, M. Canonical sampling through velocity rescaling. *J Chem Phys* **2007**, 126, 14101–14107.

(14) Berendsen, H. J. C., Postma, J. P. M., van Gunsteren, W. F., a DiNola & Haak, J. R. Molecular dynamics with coupling to an external bath. *J Chem Phys* **1984**, 81, 3684–3690.

(15) Darden, T., York, D. & Pedersen, L. Particle mesh Ewald: An N log(N) method for Ewald sums in large systems. *J Chem Phys* **1993**, 98, 10089.

(16) Hess, B., Bekker, H., Berendsen, H. J. C. & Fraaije, J. G. E. M. LINCS: A linear constraint solver for molecular simulations. *J Comp Chem* **1997**, 18, 1463–1472.

(17) Abrahama, M. J., Murtolad, T., Schulzb, R., Pall, S., Smith, J.C., Hessa, B., Lindahl, E. GROMACS: High performance molecular simulations through multi-level parallelism from laptops to supercomputers. *SoftwareX* **2015**, 1–2, 19–25.

(18) Craven, G. B.; Affron, D. P.; Allen, C. E.; Matthies, S.; Greener, J. G.; Morgan, R. M. L.; Tate, E. W.; Armstrong, A.; Mann, D. J. High-Throughput Kinetic Analysis for Target-Directed Covalent Ligand Discovery. *Angew. Chemie - Int. Ed.* **2018**, 57 (19), 5257–5261.
